# Absence of ANGPTL4 in adipose tissue improves glucose tolerance and attenuates atherogenesis

**DOI:** 10.1172/jci.insight.97918

**Published:** 2018-03-22

**Authors:** Binod Aryal, Abhishek K. Singh, Xinbo Zhang, Luis Varela, Noemi Rotllan, Leigh Goedeke, Balkrishna Chaube, Joao-Paulo Camporez, Daniel F. Vatner, Tamas L. Horvath, Gerald I. Shulman, Yajaira Suárez, Carlos Fernández-Hernando

**Affiliations:** 1 Vascular Biology and Therapeutics Program,; 2 Integrative Cell Signaling and Neurobiology of Metabolism Program, Department of Comparative Medicine,; 3 Department of Internal Medicine,; 4 Department of Cellular and Molecular Physiology, and Howard Hughes Medical Institute, and; 5 Department of Pathology, Yale University School of Medicine, New Haven, Connecticut, USA.

**Keywords:** Metabolism, Vascular Biology, Adipose tissue, Atherosclerosis, Carbohydrate metabolism

## Abstract

Alterations in ectopic lipid deposition and circulating lipids are major risk factors for developing cardiometabolic diseases. Angiopoietin-like protein 4 (ANGPTL4), a protein that inhibits lipoprotein lipase (LPL), controls fatty acid (FA) uptake in adipose and oxidative tissues and regulates circulating triacylglycerol-rich (TAG-rich) lipoproteins. Unfortunately, global depletion of ANGPTL4 results in severe metabolic abnormalities, inflammation, and fibrosis when mice are fed a high-fat diet (HFD), limiting our understanding of the contribution of ANGPTL4 in metabolic disorders. Here, we demonstrate that genetic ablation of ANGPTL4 in adipose tissue (AT) results in enhanced LPL activity, rapid clearance of circulating TAGs, increased AT lipolysis and FA oxidation, and decreased FA synthesis in AT. Most importantly, we found that absence of ANGPTL4 in AT prevents excessive ectopic lipid deposition in the liver and muscle, reducing novel PKC (nPKC) membrane translocation and enhancing insulin signaling. As a result, we observed a remarkable improvement in glucose tolerance in short-term HFD-fed AT-specific *Angptl4*-KO mice. Finally, lack of ANGPTL4 in AT enhances the clearance of proatherogenic lipoproteins, attenuates inflammation, and reduces atherosclerosis. Together, these findings uncovered an essential role of AT ANGPTL4 in regulating peripheral lipid deposition, influencing whole-body lipid and glucose metabolism and the progression of atherosclerosis.

## Abstract 2

ANGPTL4 in adipose tissue enhances circulating triglyceride clearance and glucose tolerance and attenuates ectopic lipid deposition and atherogenesis.

## Introduction

The adipose tissue (AT) of mammals has evolved as a preferred site for storage of excess calories in the form of triacylglycerol (TAG), and also as a critical endocrine organ secreting numerous factors that regulate whole-body metabolic homeostasis (1–3). The increasingly global epidemic of obesity is associated with numerous comorbidities, including dyslipidemia, cardiovascular diseases (CVDs), insulin resistance (IR), and type 2 diabetes (4, 5). Although the link between AT dysfunction and IR in different metabolic tissues has been extensively studied, the underlying mechanisms for these processes are incompletely understood. One prevailing hypothesis suggests that the imbalance between energy intake and expenditure leads to accumulation of diacylglycerol (DAG), which in turn promotes IR by enhancing the activation of protein kinase Cε (PKCε) and PKCθ in liver and muscle, respectively, leading to impaired insulin signaling (6, 7). Alternatively, accumulation of ceramides has also been proposed to mediate lipid-induced liver and muscle IR (8). Uptake of circulating lipids is regulated by lipoprotein lipase (LPL), which catalyzes the hydrolysis of TAGs from TAG-rich lipoproteins (TRLs) into glycerol and fatty acids (FAs), which are delivered to a number of metabolic tissues for storage or utilization (9, 10). Imbalance between excessive lipid uptake and reduced oxidation leads to the ectopic accumulation of lipids, resulting in IR and type 2 diabetes, two of the major risk factors of CVD (6).

LPL activity is regulated at the posttranscriptional level by ANGPTL proteins (ANGPTL3, ANGPTL4, and ANGPTL8) (11–14). ANGPTL proteins interact directly with LPL, inhibiting its dimerization and function (15, 16). The importance of ANGPTL4 in regulating TAG clearance has been demonstrated in numerous studies (14, 17). Overexpression of ANGPTL4 in mice significantly increases circulating TAG levels, in concert with a decrease in LPL activity in post-heparin plasma (PHP) and tissues (18). Conversely, global depletion of ANGPTL4 results in pronounced hypotriglyceridemia with concomitantly elevated LPL activity in tissues and PHP (19). ANGPTL4 is a secreted protein and it is thought that it can act both as a paracrine factor regulating LPL activity in other tissues as well as an autocrine factor inhibiting LPL activity in the tissue of its origin (17). However, to the best of our knowledge this possibility has never been tested because of the lack of tissue-specific knockout mouse models.

A number of genetic studies have revealed that increased plasma TAG concentrations are a risk factor for CVD (20). SNPs in *LPL* and posttranscriptional regulators of LPL including *ANGPTL4*, *APOA5*, *APOC3*, and *ANGPTL3* that modulate plasma TAGs have been persuasively linked to coronary artery disease (CAD) (21–26). For instance, the E40K missense mutation in the *ANGPTL4* gene results in reduced plasma TAG levels and confers decreased risk for CAD (27–30). However, the magnitude of the reduction in the risk of CAD as a result of inactivating mutations may be greater than would be expected from circulating TAG effects alone, given previous observations of a reduction of approximately 0.7% to 0.8% in relative risk for every genetically associated reduction of 1 mg/dl in circulating TAGs (21). These findings suggest that inactivating mutations in the *ANGPTL4* locus are cardioprotective via as yet unexplored mechanisms. Regardless of these mechanisms, there is substantial interest in ANGPTL4 and other LPL-modulating proteins as therapeutic targets for the treatment of lipid disorders. Unfortunately, despite the antiatherogenic effects of ANGPTL4 depletion, the therapeutic potential of inhibitors has been compromised owing to unexpected side effects. ANGPTL4-deficient mice or monkeys, generated through germline deletion or through the use of monoclonal antibodies against ANGPTL4, exhibit severe metabolic and systemic abnormalities upon high-fat diet (HFD) feeding (28, 31). Therefore, the development of mouse models with tissue-specific modulation of ANGPTL4 is essential to avoid confounding effects of its global depletion, and to discriminate its autocrine and paracrine roles in the eventual outcomes in health and pathological conditions.

In the present study, we report what we believe to be a novel mouse model lacking ANGPTL4 specifically in adipose tissues (Ad-KO), the tissues where the levels of ANGPTL4 are highest. Using this model, we characterize in depth the role of adipocyte-specific ANGPTL4 in lipid metabolism, obesity, and atherosclerosis. We demonstrate that under short-term HFD feeding, ANGPTL4 deficiency improves lipid metabolism and glucose tolerance without affecting other metabolic parameters including body weight (BW) or food intake. Ad-KO mice had higher LPL activity in AT and plasma, and enhanced lipid clearance from circulating TRL to white adipose tissue (WAT) and brown adipose tissue (BAT). Interestingly, increased lipid uptake by AT was counterbalanced by increased AT lipolysis, augmented FA oxidation, and reduced FA synthesis in AT, thus limiting lipid storage in AT. Importantly, redirection of lipids into AT reduced ectopic TAG and DAG accumulation in liver and skeletal muscle, decreased novel PKC (nPKC) activation, and improved HFD-induced liver and muscle insulin signaling and glucose tolerance. Moreover, mice deficient in ANGPTL4 in AT developed smaller atherosclerotic lesions compared with WT mice due to a substantial decrease in circulating lipids and cholesterol as well as a reduction in proinflammatory cytokines and endothelial cell (EC) inflammation. Overall, these results highlight the importance of AT-specific ANGPTL4 in lipid and glucose metabolism and development of cardiometabolic disorders.

## Results

### Generation of Ad-KO mice.

**Figure 1:**
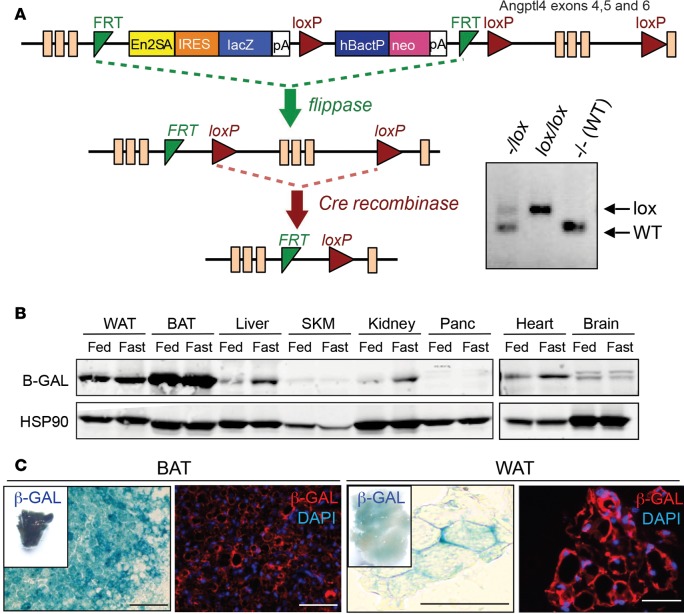
Generation of ANGPTL4 conditional deficient mice. (**A**) Schematic diagram of the inserted knockout-first allele. The cassette is composed of a short flippase recombination enzyme (Flp)-recognition target (FRT), reporter, and a Cre recombinase recognition target (loxP). The first FRT site is followed by the reporter, which is a reading frame–independent LacZ gene trap cassette: splice acceptor of mouse En2 exon 2 (En2-SA), the internal ribosome entry site from encephalomyocarditis virus (ECMV IRES), *Escherichia* *coli* lacZ gene encoding the reporter enzyme β-gal (lacZ), and simian virus 40 polyadenylation signal (pA). The first loxP site is followed by the neomycin selection cassette that is composed of the human β-actin promoter (hBactP) driving the neomycin resistance gene, pA, a second FRT site, and a second loxP site. A third loxP site is inserted downstream of the targeted exon. *Angptl4* exons 4–6 are flanked by loxP sites. Mice with the floxed allele were generated by crossing the knockout-first mice with flp recombinase-deleter mice. Subsequently, these mice were bred with mice expressing Cre recombinase to produce tissue-specific ANGPTL4-knockout mice. Genotyping from *Angptl4^fl/fl^* mice showing bands from one, both, or none of the alleles floxed. (**B**) Representative Western blot analysis showing β-gal expression from different tissues from knockout-first allele in fed and fasted conditions. SKM, skeletal muscle. (**C**) Immunostaining showing β-gal expression in brown (BAT) and white adipose tissue (WAT). Inset shows whole-mount staining and magnification shows cross sections stained with β-gal. Scale bars: 100 μm.

**Figure 2:**
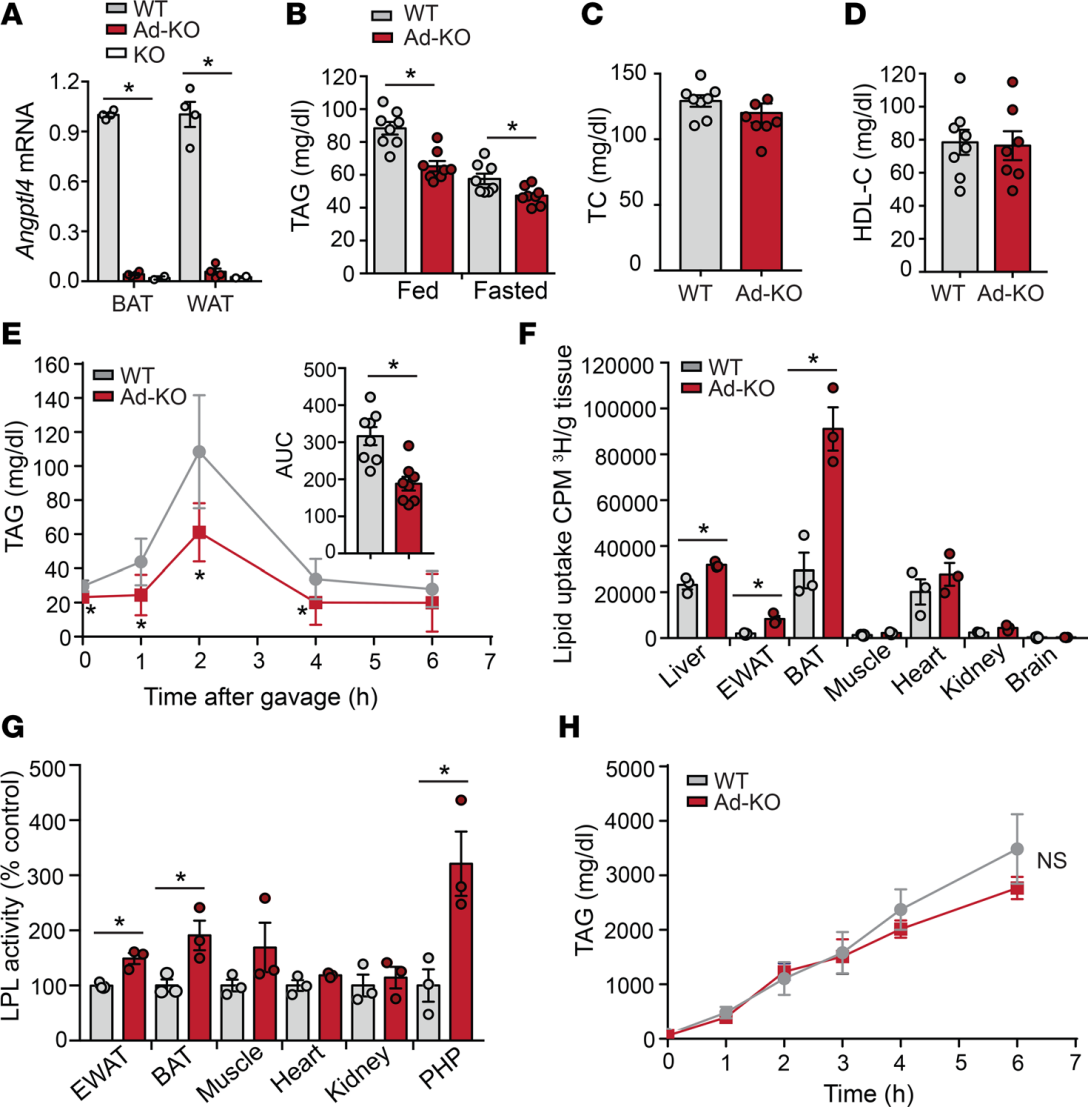
Absence of ANGPTL4 in adipose tissue (AT) enhances plasma TAG clearance and tissue lipid uptake. (**A**) mRNA expression of *Angptl4* in brown (BAT) and white adipose (WAT) tissues from WT, AT-specific *Angptl4*-knockout (Ad-KO), and *Angptl4^–/–^* mice (*n* = 4). (**B**) Fed and fasted plasma triacylglycerol (TAG) levels in WT and Ad-KO mice (*n* = 8). (**C** and **D**) Total cholesterol (TC) and HDL-C levels in plasma of WT and Ad-KO mice fasted overnight (*n* = 7–8). (**E**) Oral lipid tolerance test showing the clearance of TAGs from the plasma of fasted WT and Ad-KO mice that were fasted for 4 hours followed by an oral gavage of olive oil (*n* = 8). (**F**) Radioactivity incorporation in indicated tissues after 2 hours of oral gavage of [^3^H]-labeled triolein in WT or Ad-KO mice fasted for 6 hours (*n* = 3). (**G**) Lipoprotein lipase (LPL) activity in the indicated tissues isolated from WT and Ad-KO mice. For plasma LPL activity, blood was drawn 5 minutes after heparin injection (*n* = 3). (**H**) Plasma TAG levels from WT and Ad-KO mice fasted overnight and treated with the LPL inhibitor poloxamer 407 to inhibit lipolysis of TAG-rich lipoprotein particles (*n* = 3). All data represent the mean ± SEM. *P ≤ 0.05 comparing Ad-KO with WT mice using unpaired *t* test. NS, not significant.

To understand the role of tissue-specific ANGPTL4 in metabolic processes, we generated conditional knockout mice. We obtained ANGPTL4-mutant mice from the EUCOMM/KOMP repository, which was generated by the knockout-first strategy (32). As shown in Figure 1A, a cassette containing the mouse En2 splicing acceptor (En2SA), LacZ, and promoter-driven neomycin resistance gene (Neo) was inserted in the *Angptl4* gene. The initial allele (tm1a) is predicted to generate a null allele through splicing to the LacZ trapping element. Mice with conditional alleles (*Angptl4^fl/fl^*) were generated by removal of the gene trap cassette using Flp recombinase. These mice were cross-bred with transgenic mice in which Cre recombinase was expressed in adipocytes under the control of the *Adipoq* gene promoter (*Adipoq^CRE^*) to specifically ablate ANGPTL4 expression in AT. Since the expression of the LacZ reporter gene is regulated by the ANGPTL4 promoter gene in the original tm1a mice, the expression of ANGPTL4 was detected using an antibody against β-galactosidase (β-gal). As shown in Figure 1, B and C, β-gal was expressed abundantly in BAT and WAT and was further induced by fasting in WAT, BAT, heart, liver, and kidney. Generation of Ad-KO mice was confirmed by checking *Angptl4* mRNA levels in AT. *Angptl4* expression in WAT and BAT was completely ablated in Ad-KO mice, comparable to the levels in *Angptl4^–/–^* mice (Figure 2A). *Angptl4* is induced by fasting in a tissue-specific manner (17). As expected, expression of *Angptl4* in other tissues including liver and brain (data not shown) was not affected in Ad-KO mice (Supplemental Figure 1A; supplemental material available online with this article; https://doi.org/10.1172/jci.insight.97918DS1). In addition, expression of *Lpl* and other members of the ANGPTL family including *Angptl8* was not altered in AT from ANGPTL4 deletion (Supplemental Figure 1, B–D). *Angptl3* expression was not detected in WAT or BAT of Ad-KO mice irrespective of nutritional status.

### Absence of ANGPTL4 in WAT and BAT enhanced plasma TAG clearance and tissue lipid uptake.

To understand the whole-body effects of adipocyte-specific deletion of ANGPTL4, we assessed circulating lipids in 2-month-old WT and Ad-KO mice fed a chow diet (CD). Interestingly, we found that absence of ANGPTL4 in AT markedly reduced circulating TAGs under fed and fasting conditions (Figure 2B). We did not observe differences in circulating total cholesterol (TC) and high-density lipoprotein cholesterol (HDL-C) (Figure 2, C and D). We predicted that the hypotriglyceridemia in Ad-KO mice was due to increased TRL catabolism or defective hepatic TAG secretion. To assess how ANGPTL4 in AT influences TAG clearance, we performed a fat tolerance test. As expected, we found accelerated plasma TAG clearance after olive oil gavage in Ad-KO mice as compared with WT mice (Figure 2E). To demonstrate whether ANGPTL4 enhances TAG hydrolysis and FA uptake in AT, we performed an oral fat tolerance test with olive oil mixed with [^3^H]-triolein. Organ distribution of [^3^H]-triolein–derived radioactivity 2 hours after gavage revealed a selective increase in uptake of FA into WAT and BAT (Figure 2F). Consistent with this, LPL activity was also enhanced in WAT, BAT, and in PHP (Figure 2G). Circulating TAGs are regulated by the relative rate of TRL production and the catabolism of these particles is in turn mediated by LPL. To evaluate whether plasma TAG levels were also influenced by the rate of hepatic VLDL production in Ad-KO mice, we fasted WT and Ad-KO mice overnight and treated them with poloxamer 407, a potent inhibitor of LPL. The results showed that the rate of hepatic VLDL production was similar in both groups of mice, suggesting that the reduction in circulating TAGs observed in Ad-KO mice is due to enhanced hydrolysis of TRL in AT (Figure 2H). Taken together, these results demonstrate that ANGPTL4 expression in AT controls circulating TAG clearance and FA deposition in WAT and BAT.

### Genetic ablation of ANGPTL4 in AT does not influence BW but enhances plasma TAG clearance.

**Figure 3:**
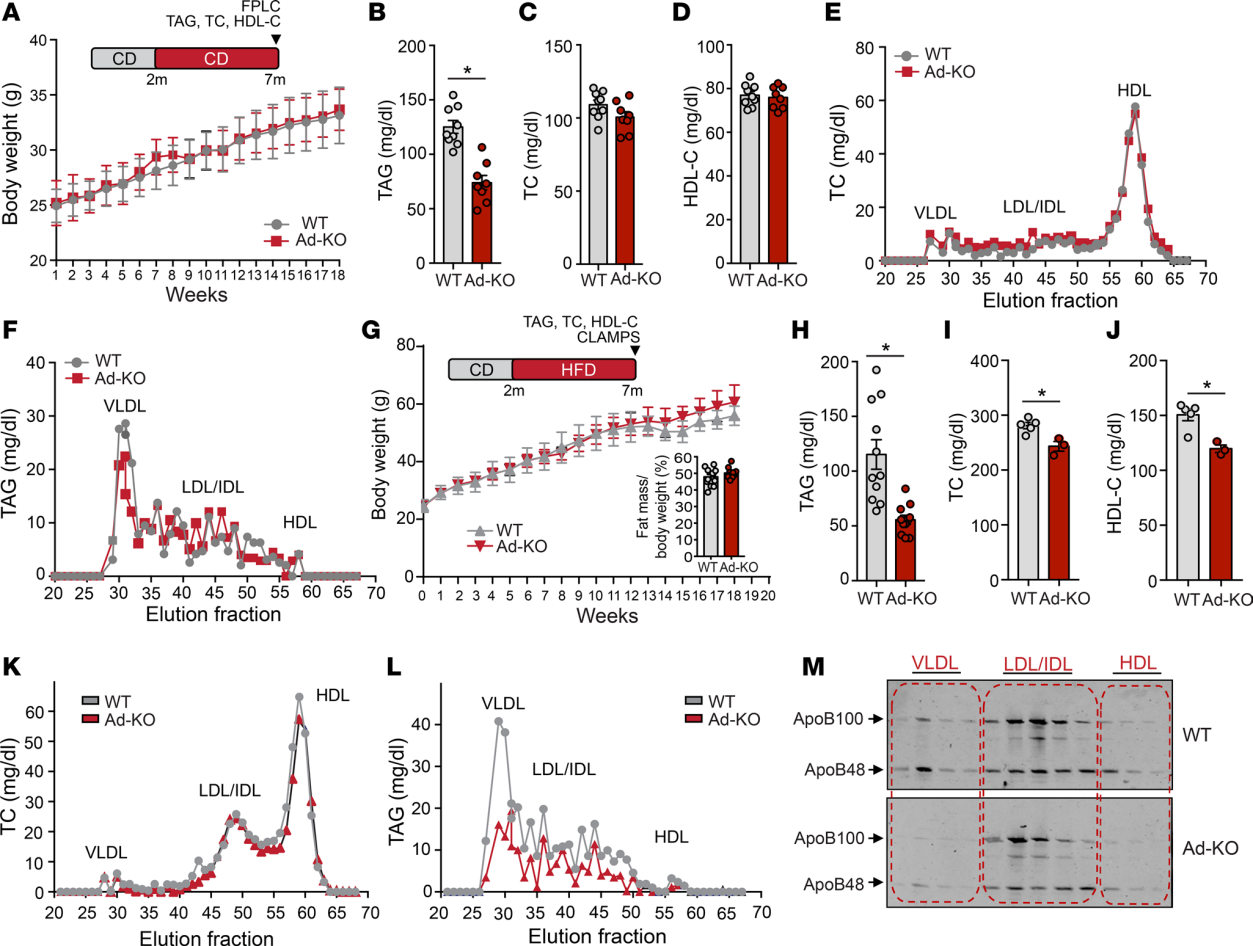
ANGPTL4 deficiency in adipose tissue (AT) affects lipid metabolism. (**A**–**D**) Body weight (**A**), plasma triacylglycerol (TAG) (**B**), total cholesterol (TC) (**C**), and high-density lipoprotein cholesterol (HDL-C) (**D**) from WT mice and in mice with AT-specific knockout of ANGPTL4 (Ad-KO) fed a chow diet (CD) for 20 weeks (*n* = 7–8). (**E** and **F**) TC (**E**) and TAG (**F**) content of FPLC-fractionated lipoproteins from pooled plasma of WT and Ad-KO mice fed a CD for 20 weeks (*n* = 7–8). (**G**) Body weight from WT and Ad-KO mice fed a high-fat diet (HFD) for 20 weeks. Insert panel shows percentage fat mass in these mice after 20 weeks on HFD (*n* = 8). (**H**–**J**) Plasma TAG (*n* = 8), (**H**) TC (*n* = 3–5) (**I**), and HDL-C (*n* = 3–5) (**J**) levels from mice fed an HFD for 20 weeks (*n* = 8). (**K** and **L**) TC (**K**) and TAG (**L**) content of FPLC-fractionated lipoproteins from pooled plasma of WT and Ad-KO mice fed an HFD for 20 weeks (*n* = 3). (**M**) Representative Western blot analysis of plasma ApoB100 and ApoB48 in the FPLC-fractionated lipoproteins. Lanes 1–13 correspond to the following pooled fractions: 1 (28–30), 2 (31–33), 3 (34–36), 4 (37–39), 5 (40–42), 6 (43–45), 7 (46–48), 8 (49–51), 9 (52–54), 10 (55–57), 11 (58–60), 12 (61–63), and 13 (64–66). All data represent the mean ± SEM. *P ≤ 0.05 comparing Ad-KO with WT mice using unpaired *t* test.

Next, we determine whether increased FA delivery to AT influences BW and metabolic function. To this end, we monitored BW weekly during 7 months in WT and Ad-KO mice fed a CD. Interestingly, we found that absence of ANGPTL4 in AT does not influence BW (Figure 3A). Similar to our previous results, circulating TAGs were significantly reduced in Ad-KO mice as compared with WT mice (Figure 3B). Plasma TC and HDL-C levels were similar in both groups of mice (Figure 3, C–E). The reduced TAG levels observed in Ad-KO mice were confirmed by measuring TAG content in the VLDL fraction isolated by fast protein liquid chromatography (FPLC) (Figure 3F).

Since ANGPTL4 regulates LPL activity and TAG clearance, we performed a separate study in which mice were fed an HFD (60% fat kcal) for 20 weeks. Similar to the results obtained in mice fed a CD, absence of ANGPTL4 in AT did not impact BW and fat mass, but significantly reduced circulating TAG concentrations (Figure 3, G and H). Moreover, plasma TC and HDL-C concentrations were significantly reduced in the Ad-KO mice (Figure 3, I and J). Lipoprotein fractionation analysis revealed a modest decrease in cholesterol (Figure 3K) and significant reduction in TAGs in the VLDL fraction (Figure 3L). Immunoblotting demonstrated a marked decrease in apoB48 and apoB100 protein content in the VLDL fractions in Ad-KO mice, indicating that the number of VLDL particles in plasma is decreased in these mice (Figure 3M).

### Metabolic characterization of Ad-KO reveals no significant alterations in BW, energy expenditure, and activity.

**Figure 4:**
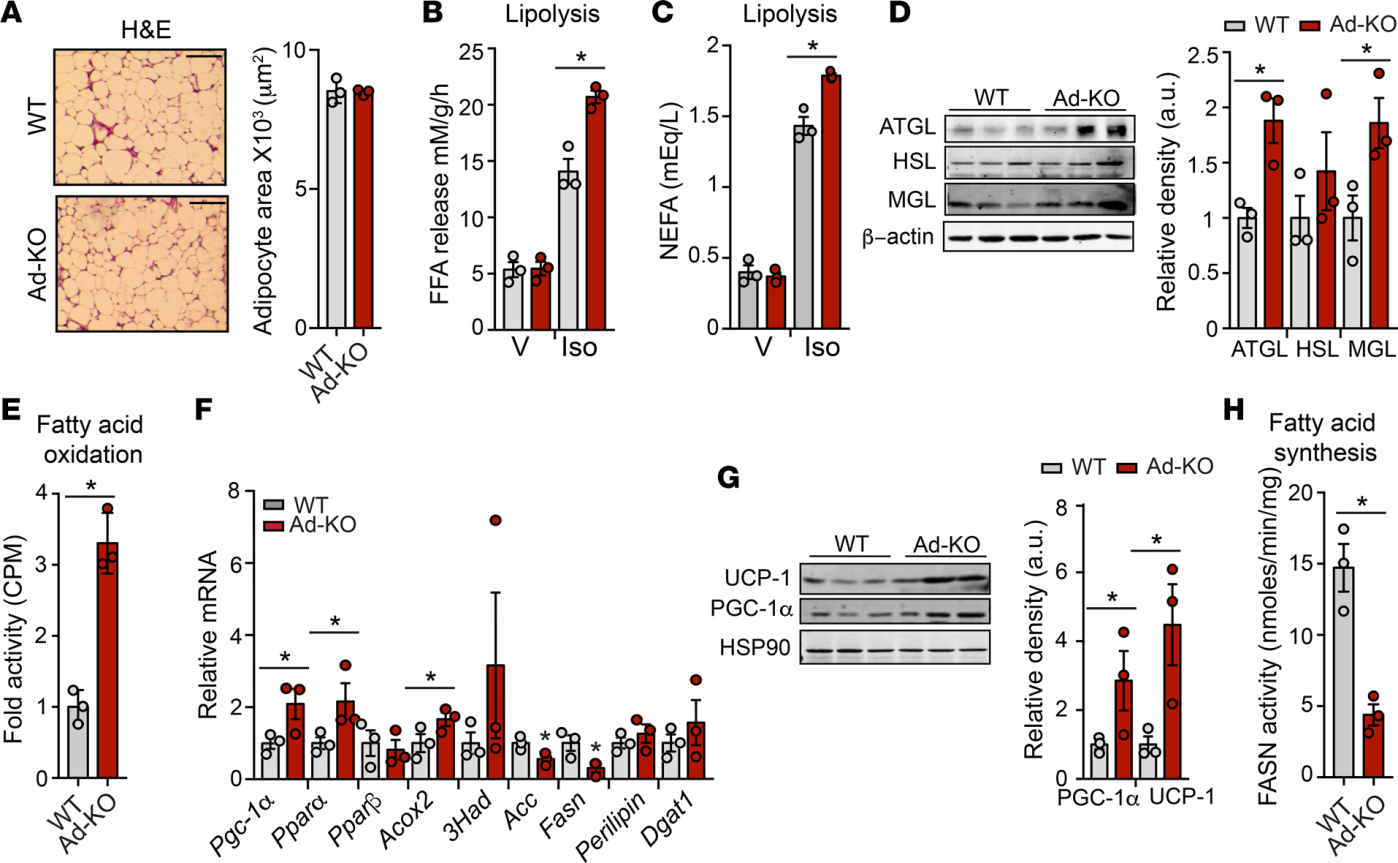
Adipose ANGPTL4 loss enhances lipolysis and oxidative metabolism but reduces endogenous lipogenesis. (**A**) Representative H&E-stained sections of white adipose tissue (WAT) isolated from WT mice and in mice with AT-specific knockout of ANGPTL4 (Ad-KO) fed a high-fat diet (HFD) for 4 weeks. Quantification of adipocyte size (right) (*n* = 3). Scale bars: 200 μm. (**B**) Ex vivo lipolysis of WAT isolated from WT and Ad-KO mice and treated with vehicle (V) or isoproterenol (Iso) (*n* = 3). FFA, free fatty acid. (**C**) In vivo lipolysis in 8-week-old WT and Ad-KO mice (*n* = 3). NEFA, nonesterified fatty acid. (**D**) Western blot analysis of indicated proteins in WAT from WT and Ad-KO mice after 4 weeks on HFD (*n* = 3). Densitometric analysis of the blots is shown on the right panel. (**E**) Ex vivo fatty acid oxidation of WAT isolated from WT and Ad-KO mice (*n* = 3). (**F**) mRNA expression of indicated genes in WAT from WT and Ad-KO mice fed an HFD for 4 weeks (*n* = 3). (**G**) Representative Western blot analysis of indicated proteins in WAT from WT and Ad-KO mice fed an HFD for 4 weeks (*n* = 3). Densitometric analysis of the blots is shown on the right panel. PGC-1α, PPARγ coactivator 1α; UCP-1, uncoupling protein 1. (**H**) Ex vivo fatty acid synthase (FASN) activity was measured in isolated WAT of WT and Ad-KO mice (*n* = 3). All data represent the mean ± SEM. *P ≤ 0.05 comparing Ad-KO with WT mice using unpaired *t* test.

In order to evaluate the effect of Ad-KO on whole-body energy balance, we performed metabolic cage studies on WT and Ad-KO mice fed an HFD for 6 weeks. The results demonstrate no difference in food consumption in either the light or dark cycle of feeding (Supplemental Figure 2A). Furthermore, there was no difference in respiratory exchange ratio (RER), locomotor activity, or energy expenditure (Supplemental Figure 2, B–D). Moreover, despite an increase in FA uptake in AT, analysis of epididymal WAT (eWAT) revealed no difference in adipocyte size in Ad-KO mice as compared with WT mice (Figure 4A). These results suggest that ANGPTL4 might also influence AT lipolysis and/or oxidation to regulate TAG storage and AT growth. Indeed, ex vivo analysis of lipolysis in WAT from WT and Ad-KO mice demonstrated that the ability of WAT to release FA in response to the β-adrenergic receptor agonist isoproterenol was augmented in Ad-KO animals (Figure 4B). Consistent with enhanced adrenergic responsiveness of adipose lipolysis, there was an increased level of circulating FAs in Ad-KO mice in response to isoproterenol administration (Figure 4C). Moreover, the lipases responsible for mobilization of FA were assessed by immunoblotting. Adipose triglyceride lipase (ATGL) and monoacylglycerol lipase (MGL) were upregulated in WAT of Ad-KO mice, whereas hormone-sensitive lipase (HSL) levels remained unchanged (Figure 4D). Next, we hypothesized that increased availability of FA would enhance FA oxidation in Ad-KO mice. Indeed, ex vivo analysis revealed an enhancement of FA oxidation in the WAT of Ad-KO mice compared with that of WT mice (Figure 4E). In line with this, genes involved in oxidative metabolism were assessed; mRNA and protein expression of PPARγ coactivator 1α (PGC-1α) and mRNA expression of the nuclear receptor *Pparα* and acyl-CoA oxidase (*Acox*) but not *of Pparβ* were significantly upregulated in the WAT of Ad-KO mice (Figure 4, F and G). In addition, protein expression of uncoupling protein 1 (UCP-1), a downstream target of PGC-1α, was also increased in Ad-KO mice, suggesting a browning of WAT of Ad-KO mice (Figure 4G). Conversely, FA biosynthesis was significantly reduced in WAT from Ad-KO mice (Figure 4H). Accordingly, expression of *Fasn* and acetyl-CoA carboxylase (*Acc*), which are involved in FA synthesis, was significantly downregulated in Ad-KO mice (Figure 4F). Reflecting an increased influx of FA into AT and enhanced FA oxidation, the ability of Ad-KO mice to maintain body temperature improved upon acute cold exposure (Supplemental Figure 3). Together, these results show that the absence of ANGPTL4 in AT leads to altered gene expression in WAT, consistent with preferential release and oxidation and reduced synthesis of FA.

### Lack of ANGPTL4 expression in AT improves glucose tolerance.

**Figure 5:**
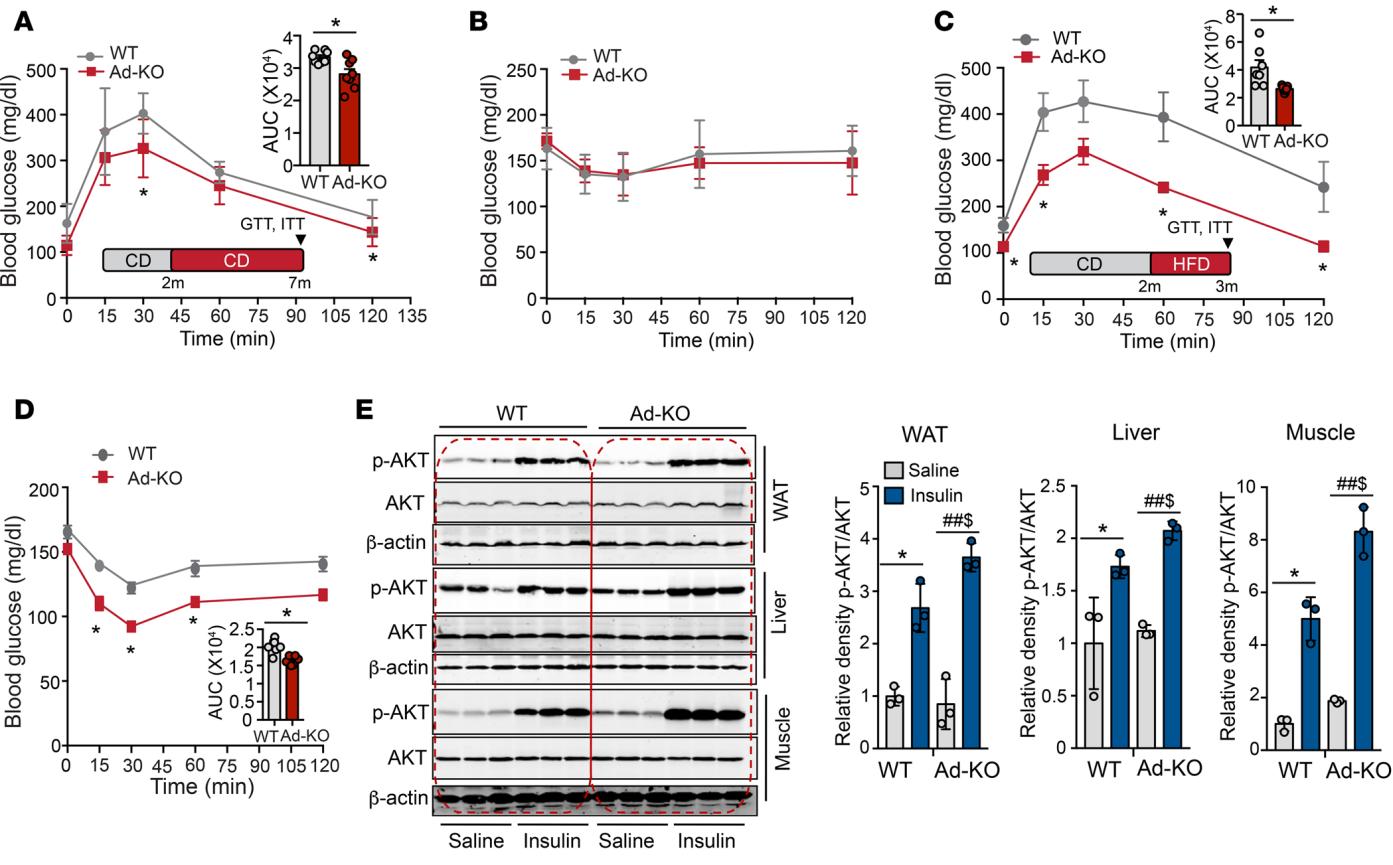
Lack of ANGPTL4 expression in adipose tissue (AT) improves glucose tolerance. (**A**–**D**) Glucose tolerance test (GTT) and insulin tolerance test (ITT) in WT mice and in mice with AT-specific knockout of ANGPTL4 (Ad-KO) mice fed a control diet (CD) (*n* = 8) (**A** and **B**) or a high-fat diet (HFD) (*n* = 7) (**C** and **D**). Inset represents the area under the curve (AUC). *P ≤ 0.05 comparing Ad-KO with WT mice using unpaired *t* test (**A**, **C**, and **D**). (**E**) Western blot analysis showing p-AKT (S^473^) and AKT levels in white adipose tissue (WAT), liver, and muscle isolated from WT and Ad-KO mice fed an HFD for 6 weeks. Densitometric analysis of the blots is shown in the right panels (*n* = 3). All data represent the mean ± SEM. *P ≤ 0.05 comparing saline with insulin within WT mice; ^##^*P* ≤ 0.05 comparing saline with insulin within Ad-KO mice; ^$^*P* ≤ 0.05 comparing Ad-KO with WT mice with insulin administration using unpaired *t* test.

We next assessed whether the reduction in circulating TAGs and enhanced lipid clearance influences glucose metabolism by performing glucose and insulin tolerance tests in CD-fed mice. Importantly, we found a significant improvement in glucose tolerance in mice lacking ANGPTL4 in AT (Figure 5A). However, we did not observe any difference in insulin tolerance between both groups of mice (Figure 5B). Since ANGPTL4 regulates LPL activity and tissue FA uptake, we next asked whether adipose ANGPTL4 deficiency is protective against lipid-induced IR. To this end, we challenged Ad-KO and WT mice with HFD for 4 weeks and assessed glucose metabolism. The results showed that absence of ANGPTL4 in AT remarkably improved glucose and insulin tolerance (Figure 5, C and D). Consistent with these results, we observed that absence of ANGPTL4 in AT leads to an improvement of insulin signaling in eWAT, which was reflected by increased phosphorylation of AKT (p-Ser473) upon insulin stimulation (Figure 5E). Interestingly, we also observed enhanced hepatic and muscle insulin signaling (Figure 5E).

**Figure 6:**
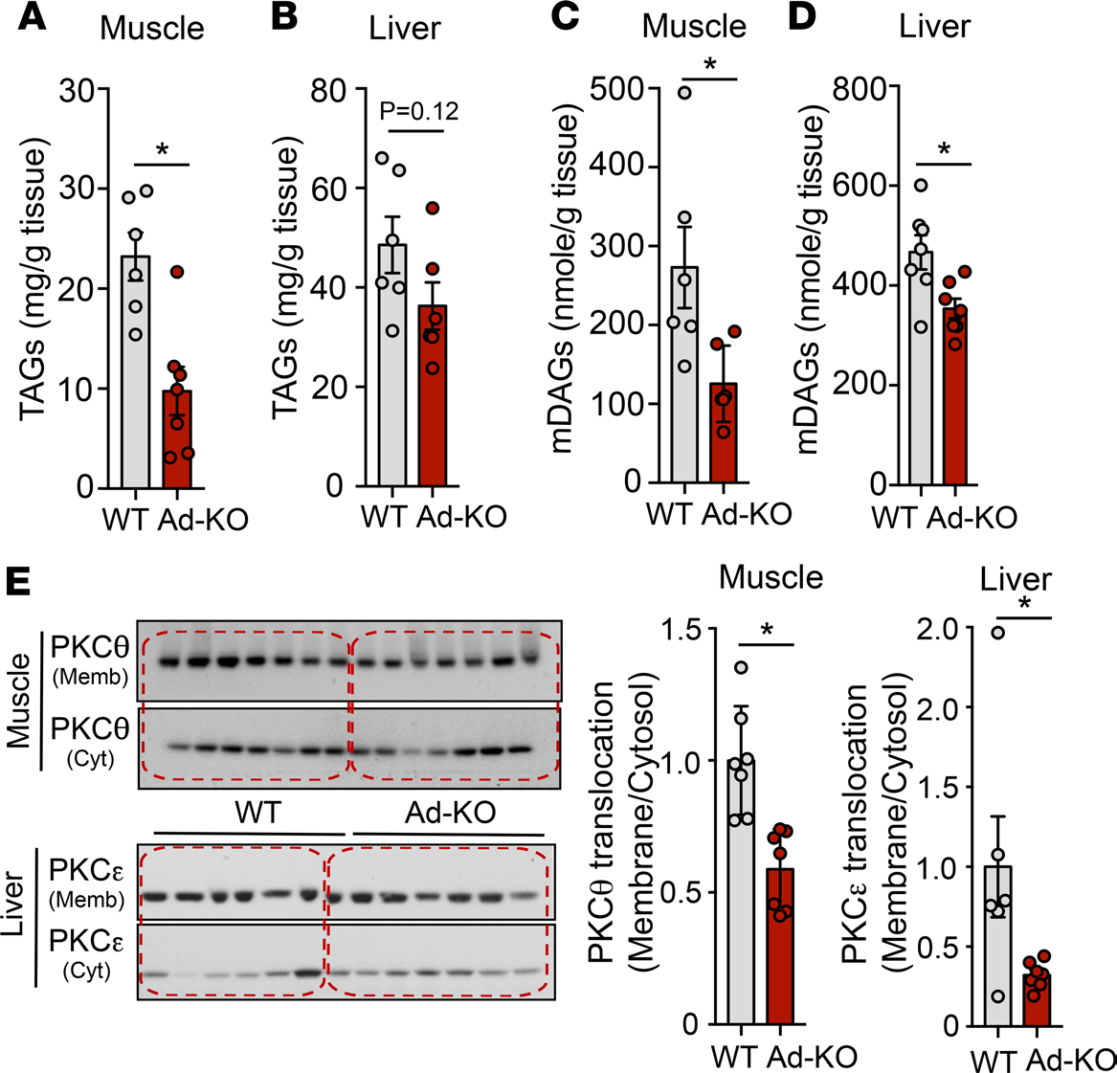
Lack of ANGPTL4 expression in adipose tissue (AT) reduces liver and muscle PKC translocation. (**A**–**D**) Muscle and liver triacylglycerol (TAG) (**A** and **B**) and diacylglycerol (DAG) (**C** and **D**) content in WT mice and in mice with AT-specific knockout of ANGPTL4 (Ad-KO) mice fed a high-fat diet (HFD) for 6 weeks and starved for 6 hours (*n* = 6). (**E**) Western blot analysis of PKCθ (upper panel) and PKCε (bottom panel) translocation in muscle and liver of WT and Ad-KO mice fed an HFD for 6 weeks and fasted for 6 h. Quantification is shown in right panels (*n* = 6–7). All data represent the mean ± SEM. *P ≤ 0.05 comparing Ad-KO with WT mice using unpaired *t* test.

The increased plasma TAG clearance by AT observed in Ad-KO mice led us to hypothesize that these mice fed an HFD for 1 month would exhibit decreased ectopic lipid content in liver and muscle. To test this hypothesis, we measured TAG content in skeletal muscle and liver of WT and Ad-KO mice. While skeletal muscle in Ad-KO mice displayed reduced TAG content as compared with WT mice (Figure 6A), we did not observe significant differences in hepatic TAG accumulation (Figure 6B). In order to understand the mechanism by which Ad-KO mice were protected from muscle and hepatic IR, we assessed the putative mediators of lipid-induced IR: DAGs (33) and ceramides (34). Of note, skeletal muscle and hepatic DAG accumulation was significantly reduced in Ad-KO mice (Figure 6, C and D). DAG-mediated activation of PKCθ and PKCε activity has been shown to mediate lipid-induced IR in skeletal muscle and liver, respectively (33). Therefore, we assessed membrane translocation of PKCθ in muscle and PKCε in liver. In accordance with the observed reduction in DAG content, we found a marked reduction in PKCθ membrane translocation in muscle and PKCε translocation in liver in Ad-KO mice compared with WT mice (Figure 6E). In contrast, there were no differences in liver or muscle ceramide content between groups (Supplemental Figure 4).

Whole-body lipid-induced IR usually develops within 4 weeks of HFD feeding. Nevertheless, the full manifested picture of obesity develops after 16 weeks of HFD with adipocyte hyperplasia, fat deposition in the mesentery, increased fat mass, and hypertension. Therefore, we next studied whether Ad-KO mice have ameliorated IR in prolonged, 20-week administration of HFD by performing hyperinsulinemic-euglycemic clamp studies. Interestingly, we found that the glucose infusion rate needed to maintain normal glucose levels after insulin administration was similar in both groups of mice (Supplemental Figure 5A). Under hyperinsulinemic conditions, insulin-stimulated glucose disposal and insulin-mediated suppression of endogenous glucose production were not altered in Ad-KO mice compared with WT mice, indicating that absence of ANGPTL4 in adipocytes does not improve lipid-induced IR after prolonged administration of HFD (Supplemental Figure 5, B and C). These observations suggest that the beneficial effect of AT ANGPTL4 deficiency in the prevention of ectopic lipid-induced IR can be offset by long-term HFD feeding.

### Loss of ANGPTL4 in adipocytes does not influence AT inflammation.

Enhanced AT inflammation has been associated with obesity and IR (35). To determine whether absence of ANGPTL4 in AT influences AT inflammation, we fed WT and Ad-KO mice an HFD for 20 weeks and analyzed total leukocytes, macrophages, and monocytes. The results show that lack of ANGPTL4 in AT does not alter total leukocyte number (Supplemental Figure 6A) and monocyte/macrophage populations (Supplemental Figure 6, B–E), indicating that infiltration of inflammatory cells in the AT is not affected by the expression of ANGPTL4 in AT.

### ANGPTL4 deficiency in adipocytes attenuates the progression of atherosclerosis.

**Figure 7:**
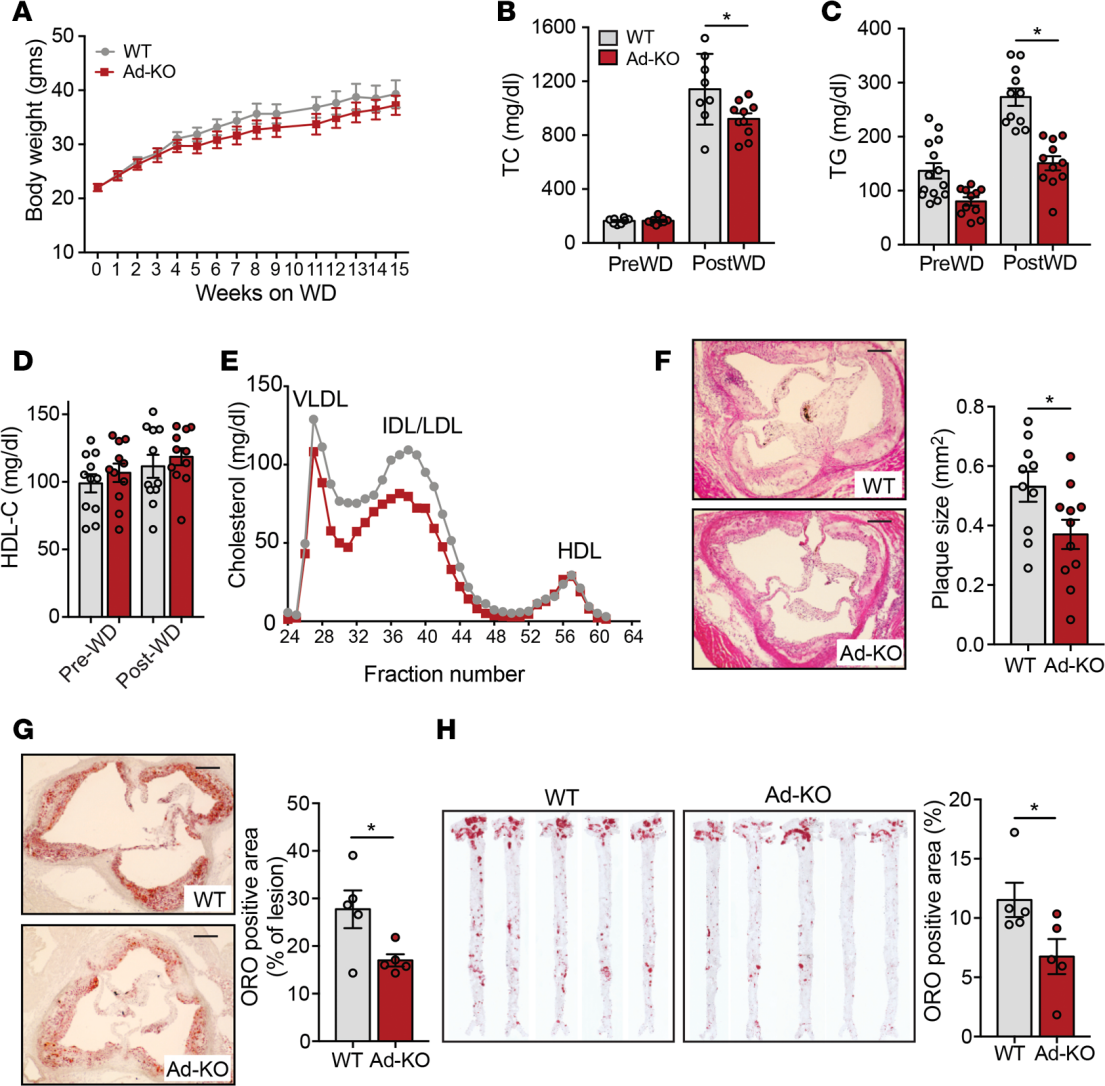
ANGPTL4 deficiency in adipose tissue (AT) attenuates atherosclerosis. (**A**–**D**) Body weight (**A**), triacylglycerols (TG) (**B**), total cholesterol (TC) (**C**), and HDL cholesterol (HDL-C) (**D**) from WT mice and in mice with AT-specific knockout of ANGPTL4 (Ad-KO) mice fed a Western diet (WD) for 16 weeks (*n* = 8). (**E**) Lipoprotein profile of pooled plasma isolated from WT and Ad-KO mice after 16 weeks on a Western diet (WD) (*n* = 5). (**F** and **G**) Representative histological analysis of cross sections of the aortic sinus isolated from WT and Ad-KO mice fed a WD for 16 weeks stained with H&E (**F**) (*n* = 10) and Oil Red O (ORO) (**G**) (*n* = 5). Quantification of the plaque size and percentage ORO area are shown in right panels. Scale bars: 200 μm. (**H**) Representative pictures from en face analysis of aortas isolated from WT and Ad-KO mice fed a WD for 16 weeks. Percentage ORO area is quantified on the right (*n* = 5). All data are the mean ± SEM. *P ≤ 0.05 comparing Ad-KO with WT mice by unpaired *t* test.

Circulating lipid and lipoprotein levels are associated with increased risk of CVD (36, 37). We have previously demonstrated that germline deletion of ANGPTL4 results in reduced circulating TAGs and atherosclerosis while also causing severe inflammation and metabolic complications (38). In contrast, hematopoietic cell–specific deletion of ANGPTL4 avoided these complications and resulted in increased atherosclerosis as a result of increased monocyte proliferation and enhanced lipid accumulation in macrophages and generation of foam cells (38). Since we observed decreased circulating TAG levels, and TC levels in Ad-KO mice on HFD, we hypothesized that ANGPTL4 depletion in AT is antiatherogenic. In order to test this hypothesis, we injected WT and Ad-KO mice with adenoviral constructs overexpressing PCSK9 (AAV8-PCSK9), an enzyme that degrades hepatic low-density lipoprotein receptor (LDLr) and increases circulating proatherogenic lipoproteins comparable to hyperlipidemia observed in the well-established atherogenic *Ldlr^–/–^* mice (39, 40). Two weeks following the injection, mice were fed a Western-type diet (WD) for 16 weeks and atherosclerosis was assessed at the end of the feeding period. There was no difference in BW between WT and Ad-KO mice throughout the study (Figure 7A). Consistent with the observations made in HFD-fed mice, Ad-KO mice had significantly less cholesterol after WD (Figure 7B). As expected, circulating TAGs were significantly reduced in Ad-KO mice compared with WT mice (Figure 7C), whereas circulating HDL-C levels were similar in the 2 groups (Figure 7D). Plasma lipoprotein fractionation using FPLC revealed that the decreased cholesterol levels in Ad-KO mice were observed in VLDL and IDL/LDL fractions (Figure 7E). Importantly, the reduced plasma lipid levels observed in Ad-KO mice correlated with a marked reduction of atherosclerotic plaque size in the aortic root (Figure 7F). In addition, the neutral lipid accumulation in the lesions was significantly attenuated in Ad-KO mice (Figure 7G). Similar results were observed when lesions in the whole aorta were assessed by en face Oil Red O staining (Figure 7H). Atherogenesis was not influenced by differences in circulating inflammatory leukocytes that were similar in WT and Ad-KO mice (Supplemental Figure 7). These results support a proatherogenic role of adipocyte-specific ANGPTL4 mediated through the modulation of circulating lipid levels and their subsequent accumulation in the plaques in atheroprone regions of large blood vessels.

**Figure 8:**
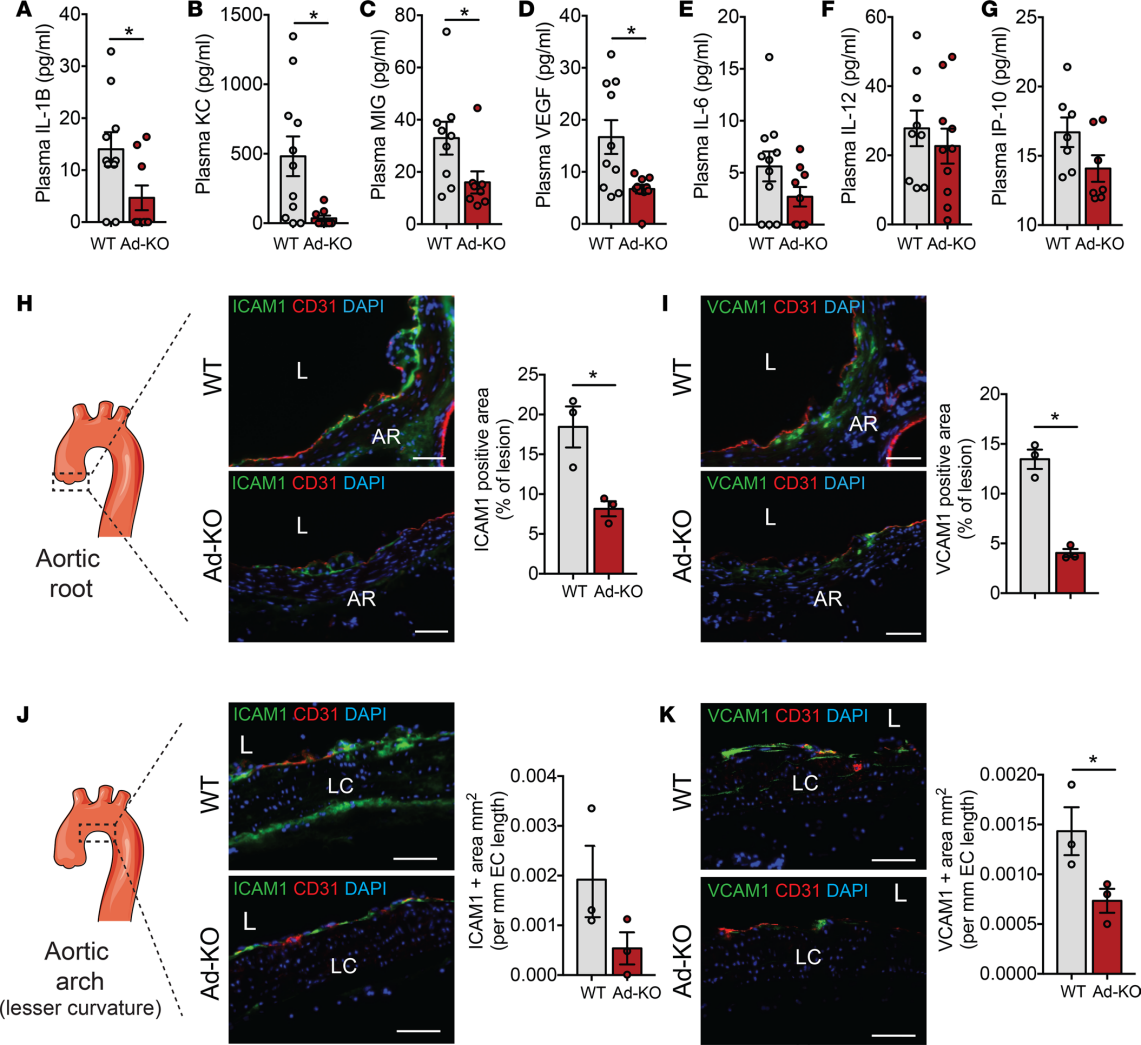
ANGPTL4 deficiency in adipose tissue (AT) reduces inflammation. (**A**–**G**) Plasma concentration of indicated cytokines from WT mice and in mice with AT-specific knockout of ANGPTL4 (Ad-KO) mice fed a Western diet (WD) for 16 weeks measured by ELISA (*n* = 7–11). (**H** and **I**) Immunostaining showing the expression of ICAM-1 (**H**) and VCAM-1 (**I**) along with CD31 in section from aortic root atherosclerotic lesions of WT and Ad-KO mice fed a WD for 4 weeks (*n* = 3). The scheme on the left shows the region from which sections were taken. Panels on the right of each image show quantification of staining expressed as percentage of lesion area. AR, aortic root; L, lesion. (**J** and **K**) Immunostaining showing the expression of ICAM-1 (**J**) and VCAM-1 (**K**) along with CD31 in a section from the lesser (LC) curvature of aortic arch of WT and Ad-KO mice fed a WD for 4 weeks (*n* = 3). The scheme on the left shows the region from which sections were taken. Panels on the right of each image shows quantification of staining expressed as positive area per unit length of endothelium. Scale bars: 62 μm (**H**–**K**). All data are the mean ± SEM. *P ≤ 0.05 by comparison with data from Ad-KO with WT mice by unpaired *t* test.

**Figure 9:**
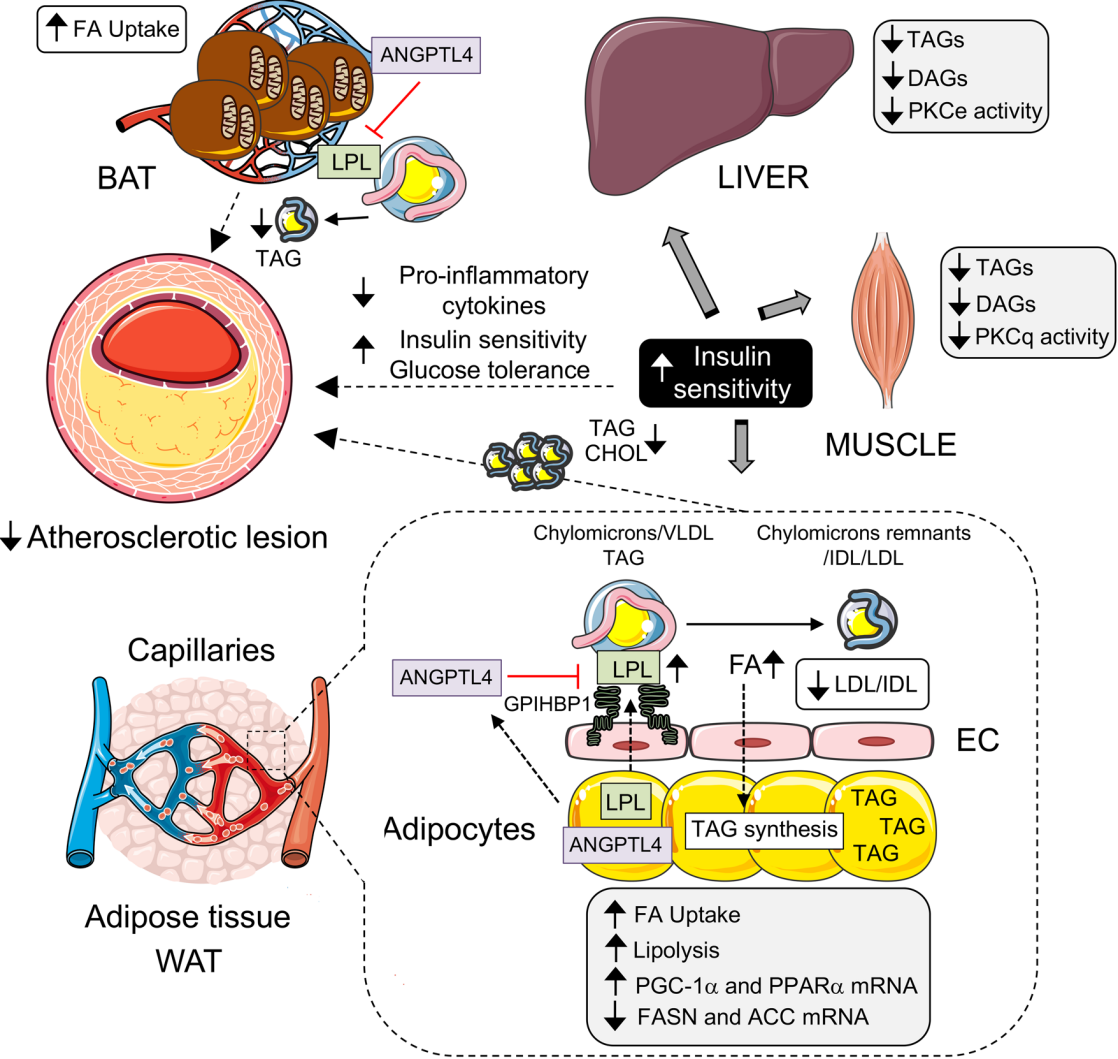
ANGPTL4 deficiency in adipose tissue (AT) improves insulin sensitivity and reduces atherosclerotic plaque burden. Schematic diagram showing the role of AT-specific ANGPTL4 in glucose and lipid metabolism and development of atherosclerosis. ANGPTL4, secreted by white (WAT) and brown adipose (BAT) tissues, binds to and inhibits lipoprotein lipase (LPL) activity and prevents triacylglycerol (TAG) hydrolysis from chylomicrons and VLDL particles. ANGPTL4 deletion from AT results in increased LPL activity, decreased circulating TAGs, and increased fatty acid (FA) uptake by AT. In addition, ANGPTL4 depletion increases lipolysis and expression of fatty acid oxidation genes while reducing expression of fatty acid synthesis genes. Concomitantly, it also results in decreased TAG, diacylglycerol (DAG), and ceramide accumulation in liver and muscles, reduces membrane translocation of PKC proteins, and increases insulin sensitivity in these organs. Additionally, absence of adipose ANGPTL4 results in reduction in lipid accumulation and size of atherosclerotic lesions. Overall, ANGPTL4 deficiency in AT improves glucose and lipid metabolism and reduces atherosclerosis. ACC, acetyl-CoA carboxylase; EC, endothelial cell; FASN, fatty acid synthase.

Levels of ANGPTL4 are correlated with inflammatory markers including C-reactive protein and IL-1β in serum from patients with type 2 diabetes and breast cancer (41, 42). In order to determine whether the differences observed in atherosclerosis in Ad-KO were mediated by differences in inflammatory mediators in addition to circulating lipids, we examined the circulating level of a panel of cytokines in plasma isolated from WT and Ad-KO mice fed a WD for 4 months. Interestingly, we found a significant reduction in circulating proinflammatory cytokines including IL-1β, KC (also known as chemokine (C-X-X) motif ligand 1 or CXCL1), and MIG (also known as CXCL9) and angiogenic factor VEGF in the plasma of Ad-KO mice compared with WT mice (Figure 8, A–D). Moreover, there was a decreasing trend in the levels of other inflammatory cytokines including IL-6, IL-12, and IP-10, although they did not reach statistical significance (Figure 8, E–G). Chemokines and inflammatory cytokines play an active role in the initiation of atherosclerosis by either recruiting leukocytes via chemotaxis or by activating ECs to induce the expression of cell adhesion molecules that in turn can recruit circulating leukocytes into the subendothelial space (36). To assess whether the differences in cytokine levels between the 2 groups account for vascular inflammation and early atherosclerosis development, we fed WT and Ad-KO mice a WD for 4 weeks after PCSK9 injection to promote atherogenesis. Then, we performed cross-sectional analysis of the lesser curvature of the aortic arch and the aortic root, which are the preferable sites of atherosclerotic plaque formation and assessed inflammation. Interestingly, Ad-KO mice had significantly reduced expression of cell adhesion molecules ICAM-1 and VCAM-1 in both sites compared with WT mice, suggesting that ANGPTL4 deficiency attenuates early vascular inflammation in proatherogenic areas (Figure 8, H–K). Our results strongly suggest that absence of adipose ANGPTL4 reduces atherosclerosis progression by decreasing circulating proatherogenic lipoproteins, inflammatory cytokines, and vascular inflammation (Figure 9).

Taken together, the results of this study elucidated the contribution of ANGPTL4 in AT in regulating lipoprotein metabolism, whole-body glucose metabolism, and atherosclerotic vascular disease using a potentially novel mouse model that lacks the expression of ANGPTL4 specifically in AT. Importantly, we described how AT ANGPTL4 modulation of lipid metabolism results in improved glucose tolerance, insulin sensitivity, and protection against the progression of atherosclerosis (Figure 9).

## Discussion

The role of ANGPTL4 in regulating lipoprotein metabolism has gained much attention during the last decade. ANGPTL4 regulates TRL catabolism, thus controlling circulating lipoprotein levels and lipid deposition in storage (AT) and oxidative tissues (heart and muscle). Despite its relevance in regulating whole-body lipid metabolism, the molecular mechanism (autocrine or paracrine) and the specific contribution of ANGPTL4 in different metabolic tissues in regulating lipid metabolism was not previously studied. In this work, we generated a mouse model lacking ANGPTL4 expression specifically in the AT, where ANGPTL4 is most highly expressed. Using these mice, we were able to identify the critical role of ANGPTL4 in AT in regulating ectopic lipid deposition, lipoprotein metabolism, glucose homeostasis, insulin sensitivity, and vascular disease. Previous studies have used either global deletion or transgenic overexpression strategies to investigate the effects of ANGPTL4 in lipid metabolism (18, 19). However, the relevance of ANGPTL4 during obesity and atherosclerosis could not be explored previously because global ANGPTL4-deficient mice fed an HFD or WD develop a severe and lethal phenotype, characterized by massive intestinal inflammation and fibrosis (31). To circumvent these complications, we generated and characterized the role of AT-derived ANGPTL4 in regulating whole-body glucose and lipid metabolism under multiple metabolic conditions. Importantly, we found that lack of ANGPTL4 in adipocytes accelerates TRL catabolism and reduces circulating TAG. These findings are consistent with multiple prior in vivo and in vitro studies that have demonstrated that ANGPTL4 inhibits LPL activity and thus regulates TAG catabolism (14, 15, 17–19). Mice overexpressing ANGPTL4 exhibited lower plasma LPL activity associated with decreased plasma TAG clearance (43). Similarly, Dewey and colleagues elegantly demonstrated that use of a monoclonal antibody against ANGPTL4 in mice and nonhuman primates reduced TAG levels due to modulation of LPL activity (28). Consistent with these data, we also observed increased plasma TAG clearance in Ad-KO mice challenged with oral lipid. This observation was associated with increased AT lipid uptake and increased LPL activity in AT and plasma, confirming that AT-specific ANGPTL4 modulates plasma TAG levels, in part, by inhibiting LPL activity.

While the impact of ANGPTL4 on lipoprotein metabolism is well established, much less is known about its potential role in glucose metabolism. Two contrasting studies identified ANGPTL4 as an important regulator of glucose homeostasis. Xu et al. demonstrated that adenovirus-mediated overexpression of ANGPTL4 improves glucose tolerance, while it induced hyperlipidemia, fatty liver, and hepatomegaly (44). In contrast, Mandard et al. reported that transgenic ANGPTL4 overexpression modestly attenuated glucose tolerance (43). This effect was enhanced when ANGPTL4-transgenic mice were fed an HFD. The latter findings are consistent with our results that show increased glucose tolerance in mice lacking ANGPTL4 in AT.

These discrepancies could have resulted from the use of different mouse models and different extent of ANGPTL4 overexpression in different tissues (liver vs. peripheral tissues). Using an alternative approach, we found that depletion of ANGPTL4 expression in AT leads to increases in plasma TAG clearance and enhanced FA uptake in AT. This increased tissue lipid uptake by AT reduced ectopic lipid (TAG and DAG) accumulation in liver and skeletal muscle, decreased nPKC activation, and prevented HFD-induced liver and muscle IR. These observations are consistent with a recent report in a dexamethasone-induced glucose intolerance model where global ANGPTL4 deficiency improved insulin sensitivity in liver (45). In this study, ANGPTL4 was suggested to dampen insulin signaling through increased ceramide levels. In our study, we did not find differences in skeletal muscle and liver ceramide content, thus dissociating changes in tissue ceramide content from IR in this model. In contrast, DAG content and nPKC activity were significantly reduced in skeletal muscle and liver from Ad-KO mice compared with WT mice and associated with improved glucose tolerance and insulin sensitivity.

The hypotriglyceridemia observed after deletion of ANGPTL4 in adipocytes might lead one to hypothesize that absence of ANGPTL4 in AT would accelerate TLR catabolism and increase lipid deposition in AT, while decreasing circulating TAGs and reducing ectopic lipid accumulation in skeletal muscle and liver. The marked decline in plasma TAGs without change in fat mass and adipocyte size despite increased FA uptake suggests that within AT of Ad-KO mice, FA were either efficiently oxidized or stored as TAG that might be further lipolyzed and oxidized, or a combination of both. Indeed, we observed an increase in adipose β-adrenergic–stimulated lipolysis and FA oxidation and a decrease in adipose FA synthesis in Ad-KO mice. These findings are consistent with studies of AT-specific LPL-deficient mice (46, 47). Numerous reports have shown that there is depleted uptake of fat in AT and a compensatory increase in de novo FA synthesis in mice lacking LPL in AT (46, 47).

The genetic loss of ANGPTL4 in mice, as well as in humans, has received considerable attention owing to its potential beneficial effects on CAD risk, especially reducing circulating TAGs which, along with circulating cholesterol, are considered as independent indicators of cardiovascular disorders including atherosclerosis (21, 48). In line with this proposition, we found that Ad-KO mice had both decreased plasma cholesterol and TAGs and reduced atherosclerosis. However, as mentioned previously, the magnitude of CAD risk reduction as a result of inactivating mutations is greater than would be expected from the circulating TAG effects alone, implicating additional mechanisms that lead to beneficial effects. Endothelial dysfunction and activation play a major role during early stages of atherosclerosis (49). Previous studies have suggested that ANGPTL4 modulates vascular permeability and inflammation, 2 processes critical for infiltration of lipoprotein particles and immune cells in the subendothelial space during atherogenesis (50–53). Interestingly, in this study, we demonstrated that deficiency of adipose ANGPTL4 lowers inflammation and decreases EC activation by lowering expression of the cell adhesion molecules ICAM-1 and VCAM-1. The attenuation in the expression of cell adhesion molecules in ECs could have resulted either indirectly from a reduction in circulating inflammatory cytokines or directly from reduced circulating ANGPTL4 in Ad-KO mice. ANGPTL4 is known to bind to integrins and other extracellular matrix proteins in ECs and trigger downstream signaling cascades (52). Whether and how ANGPTL4 binds to the cell surface receptors in ECs to regulate the expression of cell adhesion molecules and contribute to atherosclerosis requires further investigation.

In summary, we determined the role of adipose ANGPTL4 in regulating lipid and glucose metabolism and vascular inflammation and elucidate its importance in adiposity and development of atherosclerosis. We demonstrate that adipose ANGPTL4 governs plasma TAG levels by modulating LPL activity, and as a consequence, impacts associated metabolic outcomes including progression of atherosclerosis and glucose homeostasis in mice. These observations highlight the importance of this inhibitory action of ANGPTL4 and suggest that modulation of ANGPTL4 might have significant effects on TAGs and their metabolism in patients with diabetes and CVD. The findings from this study suggest that interventions capable of decreasing ANGPTL4 expression, specifically in AT, may improve glucose homeostasis and lessen atherosclerosis burden while avoiding the deleterious systemic effects observed with interventions that knock down ANGPTL4 globally.

## Methods

### Animals.

Male C57BL/6 (WT) mice were purchased from The Jackson Laboratory (JAX) and kept under constant temperature and humidity in a 12-hour controlled dark/light cycle. ANGPTL4-mutant mice containing a construct with ANGPTL4 exons 4, 5, and 6 flanked by loxP sites and lacZ reporter gene and neomycin resistance gene flanked by FRT sites were generated using a knockout-first strategy and were purchased from EUCOMM/KOMP repository. ANGPTL4-mutant mice were mated with mice expressing FLP recombinase (JAX stock 009086) to excise the neomycin resistance gene to generate mice with ANGPTL4 conditional alleles (*Angptl4^fl/fl^*). Homozygous *Angptl4^fl/fl^* mice were crossed with the transgenic mice expressing *Cre* recombinase under the control of the ADIPOQ promoter (JAX stock 010803). Offspring inheriting both the targeted allele and the *Cre* transgene (*Adipoq-Cre*- *Angptl4^fl/fl^*) were crossed with *Angptl4^fl/fl^* mice to yield homozygous *Angptl4^fl/fl^* littermates with (Ad-KO) or without (used as control for experiments) the *Cre* transgene. All mouse strains were in the B6 genetic background. Ad-KOs were verified by PCR with *Cre* primer sequences and primers flanking the 5′ homology arm of the *ANGPTL4* gene and LoxP sites using DNA extracted from their tails. All experimental mice were housed in a barrier animal facility with constant temperature and humidity in a 12-hour dark/light cycle with free access to water and food. All mice were fed a standard CD for 8–10 weeks, after which were either switched to an HFD (60% calories from fat; Research Diets D12492) for 1–20 weeks or maintained on CD.

For atherosclerosis studies, 8-week-old mice were administered a single injection containing 1.0 × 10^11^ genome copies of recombinant adeno-associated virus (AAV) encoding PCSK9 (AAV8.ApoEHCR-hAAT.D377Y-mPCK9.bGH) i.p. to induce hyperlipidemia. Two weeks after injection, accelerated atherosclerosis was induced by feeding the mice with a WD containing 1.25% cholesterol (D12108, Research Diets).

### Insulin and glucose tolerance tests.

Insulin tolerance tests were performed following a 6-hour fast by i.p. injection of 0.75 U/kg insulin. Blood glucose measurements were taken using a Contour Ultra blood glucose meter before and 15, 30, 60, and 120 minutes after injection of insulin. Blood glucose measurements were also taken at 9:00 am in fed animals and following a 16-hour fast. Glucose tolerance tests were performed by i.p. injection of glucose at a dose of 1.5 g/kg. Blood glucose was measured at 0, 15, 30, 60, and 120 minutes after injection.

### Histology, immunohistochemistry, and morphometric analyses.

Mouse hearts were perfused with PBS and put in 10 ml 4% paraformaldehyde for 4 hours. After incubation in paraformaldehyde, hearts were washed with PBS, left with PBS for 1 hour, and put in 30% sucrose overnight. Finally, hearts were embedded in OCT and frozen. Serial sections were cut at 6-μm thickness using a cryostat. Every fourth slide from the serial sections was stained with hematoxylin and eosin (H&E), and each consecutive slide was stained with Oil Red O for quantification of the atherosclerotic lesion area. Aortic lesion size of each animal was obtained by averaging the lesion areas in at least 9 sections from the same mouse. For the analysis of inflammatory molecules in atheroprone areas, 6-μm-thick sections were prepared from the aortic root in heart and from aortic arch from mice that were put on a WD for 4 months. Inflammation in plaques and ECs was assessed by staining with antibodies against ICAM-1 (1:100; BD 550287), VCAM-1 (1:100; BD 550547), and endothelial marker CD31 (1:200; Abcam ab28364).

### En face Oil Red O staining.

Thirty-five milliliters of Oil Red O stock solution (0.2% weight/volume in methanol) was mixed with 10 ml of 1 M NaOH and filtered. Aortas opened up longitudinally were briefly rinsed with 78% methanol, stained with 0.16% Oil Red O solution for 50 minutes, and then destained in 78% methanol for 5 minutes. The lesion area was quantified as a percentage of the Oil Red O–stained area in the total aorta area.

### Lipoprotein profile and lipid measurements.

Mice were fasted for 12–16 hours overnight before blood samples were collected by retro-orbital venous plexus puncture, and plasma was separated by centrifugation. HDL-C was isolated by precipitation of non–HDL-C and both HDL-C fractions and total plasma were stored at –80°C. Total plasma cholesterol and TAGs were enzymatically measured (Wako Pure Chemicals) according to the manufacturer’s instructions. The lipid distribution in plasma lipoprotein fractions was assessed by FPLC gel filtration with 2 Superose 6 HR 10/30 columns (Pharmacia Biotech).

### Plasma cytokine, chemokine, and growth factor measurement.

Plasma cytokines, chemokines, and growth factors from mice fed a WD for 4 months were measured using the Cytokine 20-Plex Mouse Panel (Thermo Fisher Scientific, LMC0006M) according to the manufacturer’s protocol.

### Fat tolerance test.

Fat tolerance test was performed as previously described (54). Briefly, mice were fasted for 4 hours beginning at 7:00 am, followed by oral gavage of 10 μl olive oil/gram of BW. Blood samples were collected from the tail vein 0, 1, 2, 4, and 6 hours after administration.

### Tissue lipid uptake.

Determination of lipid uptake in tissues was performed as previously described (55). Briefly, mice were fasted for 6 hours beginning at 7:00 am. One hour prior to administration, an emulsion was prepared by desiccation of 2 μCi [H^3^]-triolein, addition of 100 μl of mouse intralipid 20% emulsion oil, and sonication on ice for 10 minutes at 100 W, 0.5-pulse mode to generate micelles. Two hours after administration by oral gavage, mice were euthanized and plasma/tissues collected (heart, liver, kidney, WAT, BAT, and muscle). Pieces of each tissue (100–150 mg each) were weighed using a precision balance and lipids were extracted with isopropyl alcohol/hexane (2:3 v/v). The lipid layer was collected, evaporated, and [^3^H]-cholesterol radioactivity measured by liquid scintillation counting.

### LPL activity assay.

PHP and tissue LPL activity was assayed as previously described (54). PHP was obtained from fasted mice 5 minutes after i.v. injection of 10 units of heparin/kg BW. To measure total lipase activity, PHP was incubated with 10% Intralipid/[^3^H]TG emulsion as substrate. The contribution of hepatic lipase was determined by including 1 mM sodium chloride in the assay and was subtracted from the total lipase activity to estimate LPL. Heparin-releasable LPL activity in heart, skeletal muscle, kidney, WAT, and BAT was measured as previously described (54). Briefly, freshly isolated tissues were weighed and homogenized in ice-cold PBS containing 2 mg/ml FA-free BSA, 5 U/ml heparin, 5 mM EDTA, 1% Triton, and 0.1% SDS. Homogenates were centrifuged at 2,000 *g* for 15 minutes at 4°C and supernatant was collected for LPL assay. Aliquots (100 μl) of the buffer were used for the lipase assay with 100 μl of 10% Intralipid/[^3^H]-triolein emulsion for 1 hour at 25°C. The activities were normalized to starting tissue weights.

### Hepatic VLDL-TAG secretion.

To measure the hepatic VLDL-TAG production rate, overnight-fasted mice were injected i.p. with 1 g/kg BW poloxamer 407 (Sigma-Aldrich) in PBS. Blood samples were collected immediately prior to injection and at 1, 2, 3, 4, and 6 hours following injection (56). Triglyceride concentration were measured enzymatically.

### Ex vivo lipolysis.

In vitro adipose explant lipolysis assays were performed as previously described (57). Briefly, approximately 20 mg of epididymal AT explants were incubated for 2 hours at 37°C in DMEM containing 2% FA-free BSA with or without 10 μM isoproterenol. Medium samples were collected to assay for FA using a Wako NEFA-C-kit according to the manufacturer’s protocol. The level of FA was normalized to the weight of adipose explants.

### In vivo lipolysis.

In vivo lipolysis assays were performed as previously described (58). Briefly, 7-hour-fasted mice were injected i.p. with saline and 15 minutes later blood was collected from the orbital plexus. After 2 days, the 7-hour-fasted mice were injected i.p. with isoproterenol (10 mg/kg BW) and blood was collected after 15 minutes. Plasma nonesterified FA (NEFA) concentrations were measured using a Wako NEFA-C-kit according to the manufacturer’s protocol.

### FA oxidation.

FA oxidation was assayed as previously described (59). In brief, WAT was removed from anaesthetized WT and Ad-KO mice and homogenized in 5 volumes of chilled STE buffer (pH 7.4, 0.25 M sucrose, 10 mM Tris-HCl, and 1 mM EDTA). The homogenate was immediately centrifuged and the pellet was resuspended and incubated with a reaction mixture containing 0.5 mmol/l palmitate (conjugated to 7% BSA/[^14^C]palmitate at 0.4 μCi/ml) for 30 minutes. After this incubation period, this resuspended pellet–containing reaction mixture was transferred to an Eppendorf tube, the cap of which housed a Whatman filter paper disc that had been presoaked with 1 mol/l sodium hydroxide. ^14^CO_2_ trapped in the reaction mixture media was then released by acidification of media using 1 mol/l perchloric acid and gently agitating the tubes at 37°C for 1 hour. Radioactivity that had become adsorbed onto the filter disc was then quantified by liquid scintillation counting in a β-counter.

### FA synthase (FASN) activity assay.

FASN activity was determined in tissues as previously described with slight modifications (60). Briefly, WAT from Ad-KO and WT mice was homogenized in tissue homogenization buffer (0.1 M Tris, 0.1 M KCl, 350 mM EDTA, and 1 M sucrose; pH 7.5) containing protease inhibitor cocktail (Roche). Tissue extracts were clarified by centrifuging at 9,400 *g* for 10 minutes at 4°C. For determining FASN activity, BAT homogenate was added to NADPH activity buffer (100 mM potassium phosphate buffer, pH 7.5 containing 1 mM DTT, 25 μM acetyl-CoA, and 150 μM NADPH). Malonyl-CoA (50 μM) was added to the reaction buffer to initiate the reaction. Increase in the absorbance was followed at 340 nm for 10 minutes using a spectrophotometer set in kinetic mode under constant temperature (37°C). Activity of FASN is represented as nmoles NADPH consumed per minute per mg.

### Adipocyte size.

For adipocyte size quantification and CD68 staining, perigonadal AT was fixed in zinc-formalin, mounted in paraffin blocks, cut into 5-μm sections, and stained with H&E. The area of each adipocyte (in μm^2^) was measured from tracing of all adipocytes within a field of view with ImageJ software from NIH (http://rsbweb.nih.gov/ij/). The average adipocyte area for each group was derived from measurements of at least 100 adipocytes.

### Body composition and metabolic cage analysis.

Analysis of body composition was performed by Echo MRI (Echo Medical System). A Comprehensive Lab Animal Monitoring System (CLAMS; Columbus Instruments) was used to evaluate O_2_ consumption, CO_2_ production, energy expenditure, activity, and food consumption.

### Monitoring food intake.

For evaluation of food intake, mice were separated into individual cages at least 4 days prior to experiments. For determination of daily food intake mice were fed a known amount of HFD and the remaining food was measured between 4:00 and 5:00 pm daily.

### Cold tolerance test.

For acute cold measurement, mice were kept in a cold room at 4°C without food and the rectal temperature was measured using a BAT-12 microprobe thermometer (Physitemp) at the indicated time points.

### Western blot analysis.

Tissues were homogenized by manual disruption and the Bullet Blender Homogenizer. Both tissues and cells were lysed in ice-cold buffer containing 50 mM Tris–HCl, pH 7.5, 0.1% SDS, 0.1% deoxycholic acid, 0.1 mM EDTA, 0.1 mM EGTA, 1% NP-40, 5.3 mM NaF, 1.5 mM Na_4_P_2_O_7_, 1 mM orthovanadate, 1 mg/ml protease inhibitor cocktail (Roche), and 0.25 mg/ml AEBSF (Roche). Lysates were sonicated and rotated at 4°C for 1 hour before the insoluble material was removed by centrifugation at 12,000 *g* for 10 minutes. After normalizing for equal protein concentration, cell lysates were resuspended in SDS sample buffer before separation by SDS-PAGE. Following transfer of the proteins onto nitrocellulose membranes, the membranes were probed with the following antibodies: anti-ATGL (Cell Signaling Technology, 2138; 1:1,000), anti-HSL (Cell Signaling Technology, 4107; 1:1,000), AKT, p-AKT, MGL, PKCε (BD Biosciences, 610086; 1:1,000), PKCθ (Cell Signaling Technology, 13643; 1:1,000), and anti-HSP90 (BD Biosciences, 610419; 1:1,000). Protein bands were visualized using the Odyssey Infrared Imaging System (LI-COR Biotechnology) and densitometry was performed using ImageJ software. For Western blot analysis of ApoB-100 and ApoB-48 in pooled lipoprotein fractions, separation was performed in a NuPAGE Novex 4%–12% Tris-Acetate Mini Gel using 1× NuPAGE Tris-Acetate SDS running buffer (Invitrogen). Following overnight transfer of proteins onto nitrocellulose membranes, the membranes were blocked in 5% (w/v) nonfat milk dissolved in wash buffer. The membranes were probed with an antibody against ApoB (Meridian, K23300R; 1:2,000) overnight at 4°C and visualized as above.

### nPKC translocation assay.

PKCε translocation was assessed in liver and PKCθ translocation in skeletal muscle of mice that were fasted for 6 hours. Membrane-associated and cytosolic fractions were prepared as previously described (61). After normalizing for equal protein concentration, cell lysates were resuspended in reducing SDS sample buffer and separated in 4%–12% Tris-glycine gels (Novex). Following a 2-hour semidry transfer onto PVDF membranes (Immobilon-P; EMD Millipore), the membranes were blocked in 5% (w/v) nonfat dry milk or 5% (w/v) BSA dissolved in wash buffer according to the manufacturer’s recommendations. Membranes were probed overnight at 4°C with the following primary antibodies diluted in blocking solution: PKCε, PKCθ, GAPDH (Cell Signaling Technology, 5174; 1:5,000), Na-K ATPase (Abcam, ab7671; 1:1,000), and calnexin (Abcam, ab13504; 1:1,000). Membranes were then washed in TBS-T and incubated for 1 hour at room temperature with HRP-conjugated secondary antibodies (Cell Signaling Technology) diluted in blocking buffer. After further washing in TBS-T, antibody binding was visualized by enhanced chemiluminescence (Thermo Fisher Scientific). Films were developed at multiple exposures and images within the linear dynamic range of signal intensity were scanned for digital analysis. Densitometric analysis of the gels was carried out using ImageJ software. The ratio of membrane PKC intensity (normalized to Na-K ATPase or calnexin intensity) to cytosolic PKC intensity (normalized to GAPDH intensity) was calculated.

### Liver and muscle lipid measurements.

Mice were fasted 6 hours before liver and muscle collection. Liver and muscle TAGs were extracted by the method of Bligh and Dyer (62) and measured using a colorimetric assay (Sekisui). Liver and skeletal muscle DAGs were extracted from cytosolic/lipid droplet and membrane-associated fractions and measured by LC-MS/MS essentially as described previously (63, 64). Total DAGs are reported as the sum of individual species. Liver and muscle ceramides were extracted and measured by LC-MS/MS according to previously established methods.

### RNA isolation and quantitative real-time PCR.

Total RNA from tissue was isolated using TRIzol reagent (Invitrogen) according to the manufacturer’s protocol. For mRNA expression analysis, cDNA was synthesized using iScript RT Supermix (Bio-Rad), following the manufacturer’s protocol. Quantitative real-time PCR (qRT-PCR) analysis was performed in duplicate using SsoFast EvaGreen Supermix (Bio-Rad) on an iCycler Real-Time Detection System (Eppendorf). The mRNA levels were normalized to 18S.

### Statistics.

Animal sample size for each study was chosen based on literature documentation of similar well-characterized experiments. The number of animals used in each study is listed in the figure legends. In vitro experiments were routinely repeated at least 3 times unless otherwise noted. No inclusion or exclusion criteria were used and studies were not blinded to investigators or formally randomized. Data are expressed as average ± SEM. Statistical differences were measured using an unpaired 2-sided Student’s *t* test, or 1-way ANOVA with Bonferroni’s correction for multiple comparisons. Normality was checked using the Kolmogorov-Smirnov test. A nonparametric test (Mann-Whitney) was used when data did not pass the normality test. A value of *P* ≤ 0.05 was considered statistically significant. Data analysis was performed using GraphPad Prism software version 7.

### Study approval.

All of the experiments were approved by the Institutional Animal Care Use Committee of Yale University School of Medicine.

## Author contributions

BA, AKS, YS, and CFH conceived and designed the study and wrote the manuscript. BA, AKS, LV, XZ, NR, LG, BC, JPC, and DFV performed experiments and analyzed data. TLH and GIS assisted with experimental design and data interpretation.

## Supplementary Material

Supplemental data

## Version 1 03/22/2018

Electronic publication
