# Supplementary material for: Absence of ANGPTL4 in adipose tissue improves glucose tolerance and attenuates atherogenesis
Source: JCI Insight. 2018 Mar 22;3(6):e97918. doi: 10.1172/jci.insight.97918 (PMC5926923; doi:10.1172/jci.insight.97918)
Supplement: Supplemental data [file jciinsight-3-97918-s001.pdf]

# Absence of ANGPTL4 in adipose tissue improves glucose tolerance and attenuates atherogenesis

Binod Aryal[1,2,*], Abhishek K. Singh[1,2,*], Xinbo Zhang[1,2], Luis Varela[2], Noemi Rotllan[1,2], Leigh Goedeke[3], Joao-Paulo Camporez[3], Daniel F. Vatner[3], Tamas L. Horvath[2], Gerald I Shulman[3,4], Yajaira Suárez[1,2,5,#] and Fernández-Hernando C[1,2,5,#].

[1]Vascular Biology and Therapeutics Program, Yale University School of Medicine, New Haven, Connecticut, USA.

[2]Integrative Cell Signaling and Neurobiology of Metabolism Program, Department of Comparative Medicine, Yale University School of Medicine, New Haven, Connecticut, USA.

[3]Department of Internal Medicine, Yale University School of Medicine, New Haven, Connecticut, USA.

[4]Department of Cellular and Molecular Physiology, and Howard Hughes Medical Institute, Yale University School of Medicine, New Haven, Connecticut, USA.

[5]Department of Pathology, Yale University School of Medicine, New Haven, Connecticut, USA.

**Running Title:** ANGPTL4 in cardiometabolic diseases.

## SUPPLEMENTAL INFORMATION

## SUPPLEMENTAL MATERIALS AND METHODS

### Hyperinsulinemic-euglycemic clamp studies

Hyperinsulinemic-euglycemic clamps were performed in chronically catheterized awake mice as previously described (Jurczak et al., 2012). A jugular venous catheter was implanted 6 to 7 d before the studies were performed. To assess basal whole-body glucose turnover, [3-$^{3}$H]-glucose (HPLC purified) (PerkinElmer Life Sciences, Waltham, MA, USA) was infused at a rate of 0.05 μCi/min for 120 min into the jugular catheter after with-holding food overnight. After the basal period, hyperinsulinemic-euglycemic clamps were conducted for 140 min with a 3-min primed infusion of insulin [10 mU/(kg-min)] and [3-$^{3}$H]-glucose (0.24μCi/min), followed by a continuous [2.5 mU/(kg-min)] infusion of human insulin (Novolin; Novo Nordisk, Bagsværd, Denmark) and [3-$^{3}$H]-glucose (0.1μCi/min), and a variable infusion of 20% dextrose. A 10-□Ci bolus of 2-deoxy-d-[1-$^{14}$C] glucose (PerkinElmer) was injected after 85 min to

determine insulin-stimulated tissue glucose uptake. Plasma samples were obtained from the tip of the tail at 0, 25, 45, 65, 80, 90, 100, 110, 120, 130, and 140 min. The tail cut was made at least 2 h before the first blood sample was taken to allow for acclimatization, according to standard operating procedures. Mice received an intravenous artificial plasma solution (115 mM NaCl, 5.9 mM KCl, 1.2 mM $MgCl_2$-6$H_2O$, 1.2 mM $NaH_2PO_4$-$H_2O$, 1.2 mM $Na_2SO_4$, 2.5 mM CaCl-2$H_2O$, 25 mM $NaHCO_3$, and 4% BSA [pH 7.4]) at a rate of 4.2ml/min during the insulin-stimulated period of the clamp to compensate for volume loss secondary to blood sampling. At the end of the clamps, mice were anesthetized with sodium pentobarbital injection (150 mg/kg), and all tissues taken were freeze-clamped in liquid nitrogen and stored at -80°C for subsequent use.

**Circulating leukocyte analysis**

Blood was collected by retro-orbital puncture in heparinized microhematocrit capillary tubes. Measurement of total circulating numbers of blood leukocytes was performed using a HEMAVET system. For further FACs analysis, erythrocytes were lysed with ACK lysis buffer (155 mM ammonium chloride, 10 mM potassium bicarbonate, and 0.01 mM EDTA, pH 7.4). White blood cells were resuspended in 3% fetal bovine serum in PBS, blocked with 2 mg/ml FcgRII/III, then stained with a cocktail of antibodies. Monocytes were identified as $CD115^{hi}$ and subsets as Ly6-$C^{hi}$ and Ly6-$C^{lo}$. The following antibodies were used (all from BioLegend, San Diego, CA, USA): FITC-Ly6-C (AL-21), PE-CD115 (AFS98), and APC-Ly6-G (1A8).

**SUPPLEMENTAL FIGURE LEGENDS**

**Figure S1. Absence of ANGPTL4 in AT does not influence LPL, ANGPTL3 and ANGPTL8 expression in AT and liver.**

(**A-D**) mRNA expression of *Angptl44* (**A**), *Lpl* (**B**), *Angptl3* (**C**) and *Angptl8* (**D**) in white adipose tissue (WAT) and brown adipose tissue (BAT) and liver isolated from overnight fasted and fed WT and *Ad-KO* mice. Data represent the mean ± S.E.M. of relative expression levels normalized to fed WT mice (n=4).

**Figure S2. Loss of ANGPTL4 in WAT does not influence food intake and energy utilization.** (**A**-**D**) Food consumption (**A**), respiratory exchange ratio (RER) (**B**), locomotor activity (**C**) and energy expenditure (EE) (**D**) in WT and *Ad-KO* mice fed a HFD for 6 weeks (n=7). All data represent the mean ± SEM and * indicates P < 0.05 comparing Ad-KO with WT mice using unpaired t-test.

**Figure S3. Absence of ANGPTL4 in AT improves thermoregulation.**

Rectal temperature monitor of two-month old mice WT and *Ad-KO* mice (n=5). All data represent the mean ± SEM and * indicates P < 0.05 comparing *Ad-KO* with WT mice using unpaired t-test.

**Figure S4. Loss of ANGPTL4 in AT does not influence muscle and hepatic ceramide accumulation in mice fed a HFD for 1 month.**

Muscle (*left panel*) and liver (*right panel*) ceramide content in WT and *Ad-KO* mice fed a HFD for 4 weeks and fasted for 6 h. (n=9-10). All data represent the mean ± SEM and * indicates P < 0.05 comparing Ad-KO with WT mice using unpaired t-test.

**Figure S5. ANGPTL4 deficiency in AT does not influence whole-body insulin resistance in long-term HFD fed mice.**

(**A**) Glucose infusion rate (GIR) during hyperinsulinemic-euglycemic clamp in WT and *Ad-KO* mice fed a HFD for 20 weeks. (**B**) Whole-body glucose uptake during the hyperinsulinemic-euglycemic clamp in WT and *Ad-KO* mice fed a HFD for 20 weeks. (**C**) Endogenous glucose production (EGP) measured in the basal period and during the hyperinsulinemic-euglycemic clamp in WT and *Ad-KO* mice fed a HFD for 20 weeks. EGP suppression (percent basal) during hyperinsulinemic-euglycemic clamp. All data represent the mean ± S.E.M. (n=6-8).

**Figure S6. ANGPTL4 deficiency in AT does not influence AT inflammation.**

(**A-D**) Quantification of $CD45^+$ cells (**A**), macrophages (**B**), $Ly6C^+$ macrophages (**C**) and $Ly6C^+$ monocytes (**D**) in WAT isolated from WT and Ad-KO mice fed a HFD for 20 weeks. All data represent the mean ± S.E.M. (n=7).

**Figure S7. Absence of ANGPTL4 in AT does not affect circulating leukocytes**

(**A**) Peripheral blood counts from WT and *Ad-KO* mice injected with PCSK9-AAV and fed a WD for 12 weeks measured using Hemavet hematology analyzer (n=8-10). (**B** and **C**) Flow cytometry analysis of circulating monocytes (**B**) and B and T cells (**C**) from WT and *Ad-KO* mice injected with PCSK9-AAV and fed a WD for 12 weeks. Data represent the mean ± S.E.M. (n=6-8).

## SUPPLEMENTAL REFERENCES


Jurczak, M.J., Lee, A.H., Jornayvaz, F.R., Lee, H.Y., Birkenfeld, A.L., Guigni, B.A., Kahn, M., Samuel, V.T., Glimcher, L.H., and Shulman, G.I. (2012). Dissociation of inositol-requiring enzyme (IRE1alpha)-mediated c-Jun N-terminal kinase activation from hepatic insulin resistance in conditional X-box-binding protein-1 (XBP1) knock-out mice. J Biol Chem *287*, 2558-2567.

# Figure S1

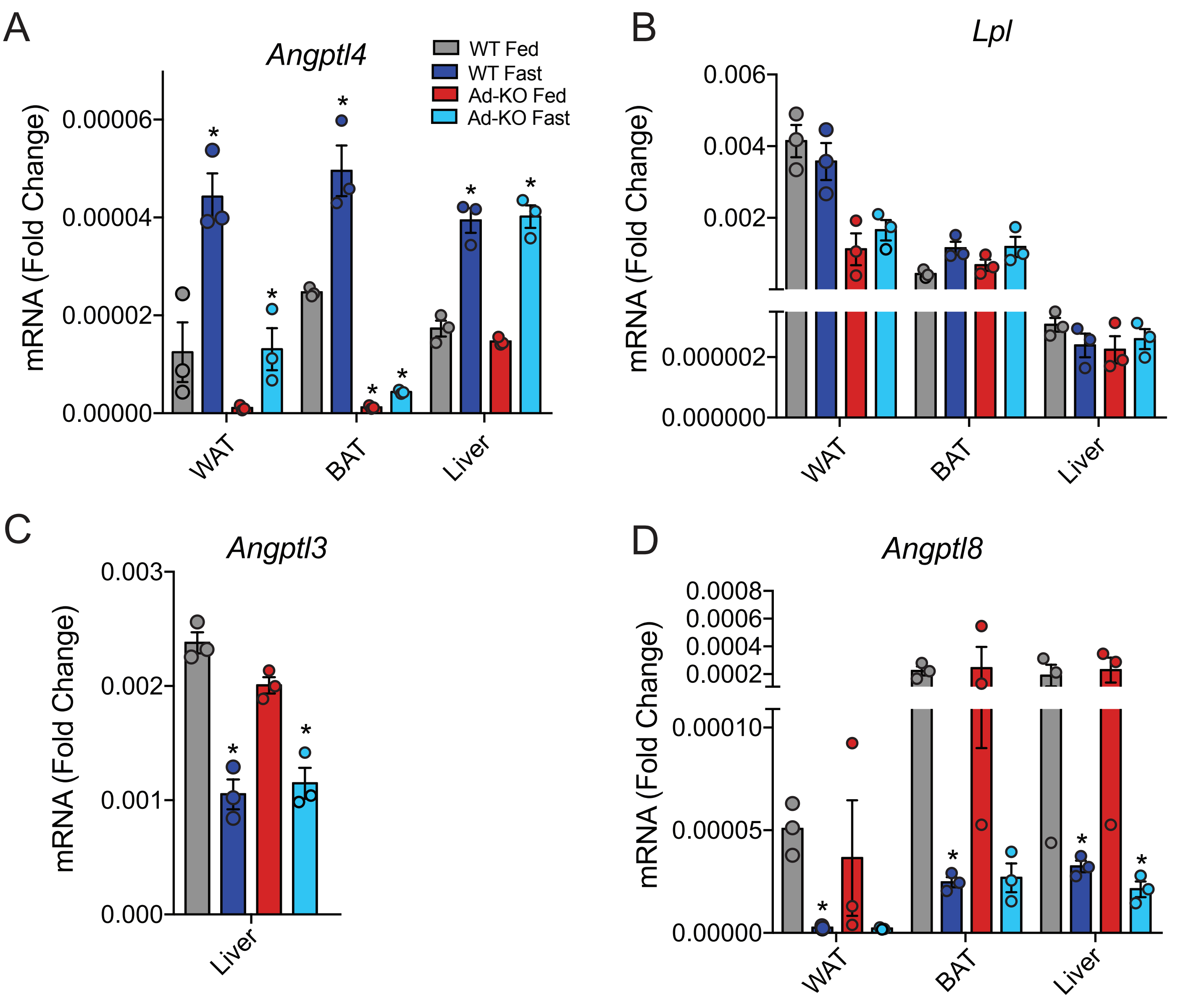

A
Angptl4
WT Fed
WT Fast
Ad-KO Fed
Ad-KO Fast
mRNA (Fold Change)
0.00006
0.00004
0.00002
0.00000
WAT
BAT
Liver
B
Lpl
0.006
0.004
0.002
0.000002
0.000000
C
Angptl3
0.003
0.002
0.001
0.000
D
Angptl8
0.0008
0.0006
0.0004
0.0002
0.00010
0.00005
0.00000

# Figure S2

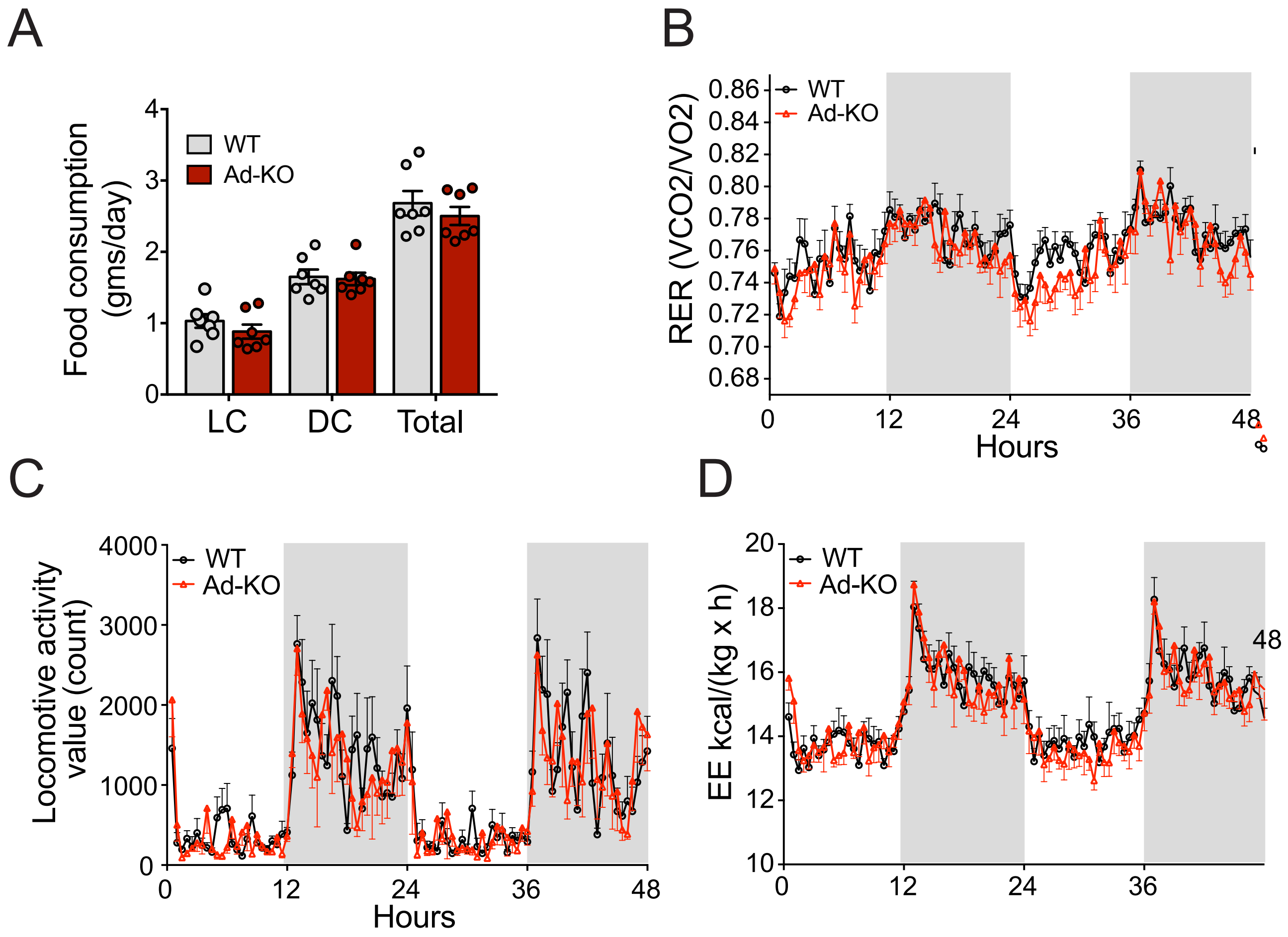

A
Food consumption (gms/day)
WT
Ad-KO
LC
DC
Total
B
RER (VCO2/VO2)
WT
Ad-KO
Hours
C
Locomotive activity value (count)
WT
Ad-KO
Hours
D
EE kcal/(kg x h)
WT
Ad-KO
48

Figure S3

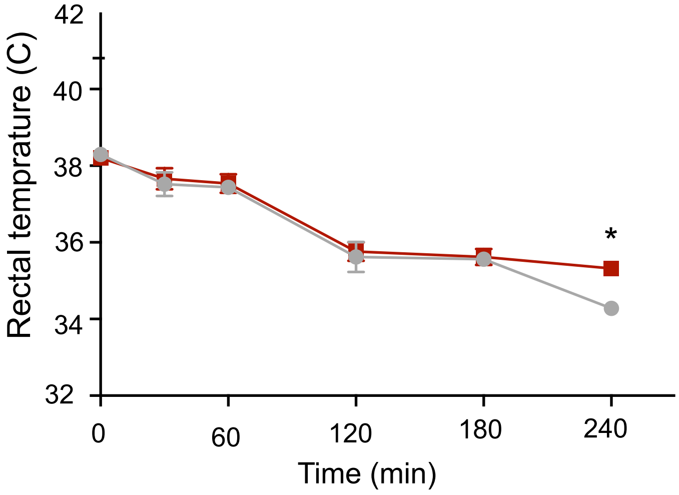

Figure S4

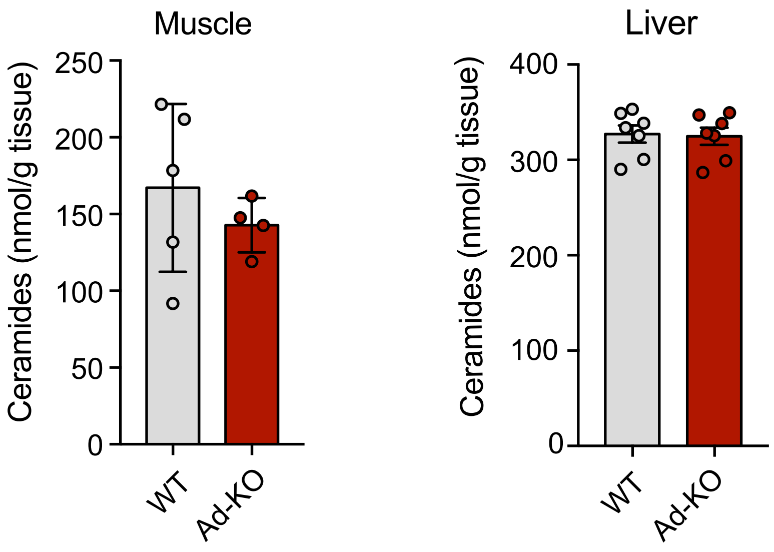

Muscle
Ceramides (nmol/g tissue)
250
200
150
100
50
0
WT
Ad-KO
Liver
Ceramides (nmol/g tissue)
400
300
200
100
0
WT
Ad-KO

Figure S5

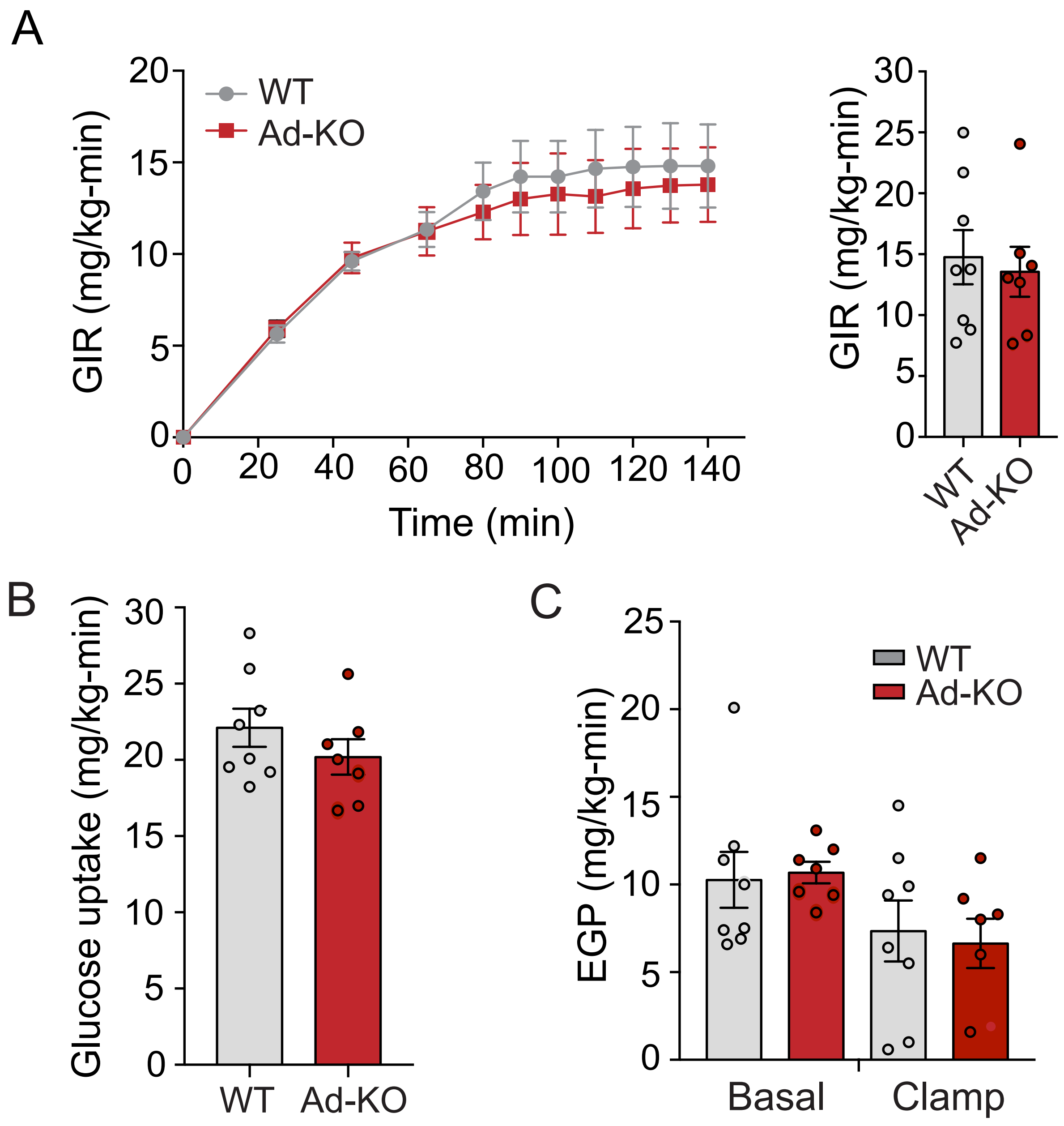
A
WT
Ad-KO
GIR (mg/kg-min)
Time (min)
0
20
40
60
80
100
120
140
0
5
10
15
20
GIR (mg/kg-min)
0
5
10
15
20
25
30
WT
Ad-KO
B
Glucose uptake (mg/kg-min)
0
5
10
15
20
25
30
WT
Ad-KO
C
WT
Ad-KO
EGP (mg/kg-min)
0
5
10
15
20
25
Basal
Clamp

# Figure S6

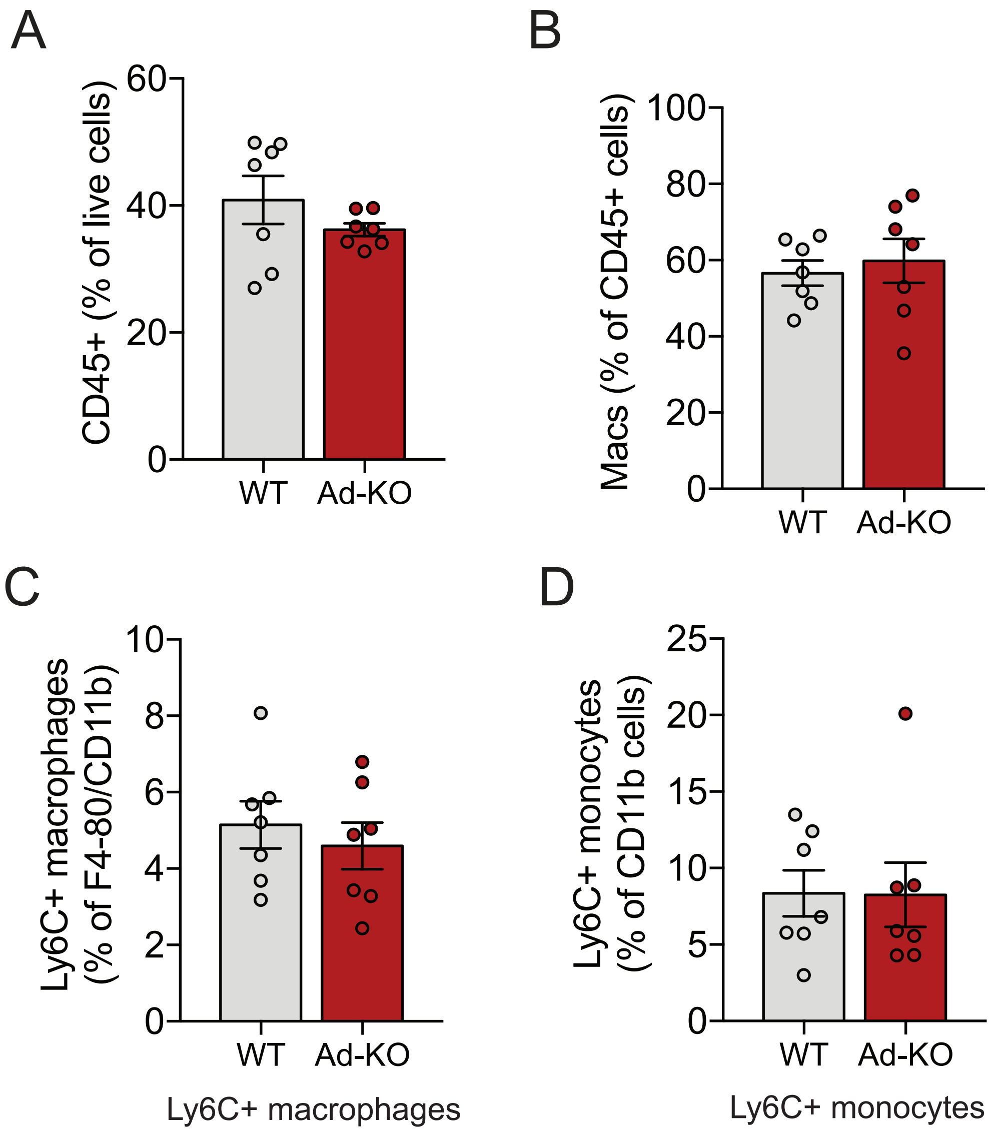
A
CD45+ (% of live cells)
0
20
40
60
WT
Ad-KO
B
Macs (% of CD45+ cells)
0
20
40
60
80
100
WT
Ad-KO
C
Ly6C+ macrophages
(% of F4-80/CD11b)
0
2
4
6
8
10
WT
Ad-KO
Ly6C+ macrophages
D
Ly6C+ monocytes
(% of CD11b cells)
0
5
10
15
20
25
WT
Ad-KO
Ly6C+ monocytes

Figure S7

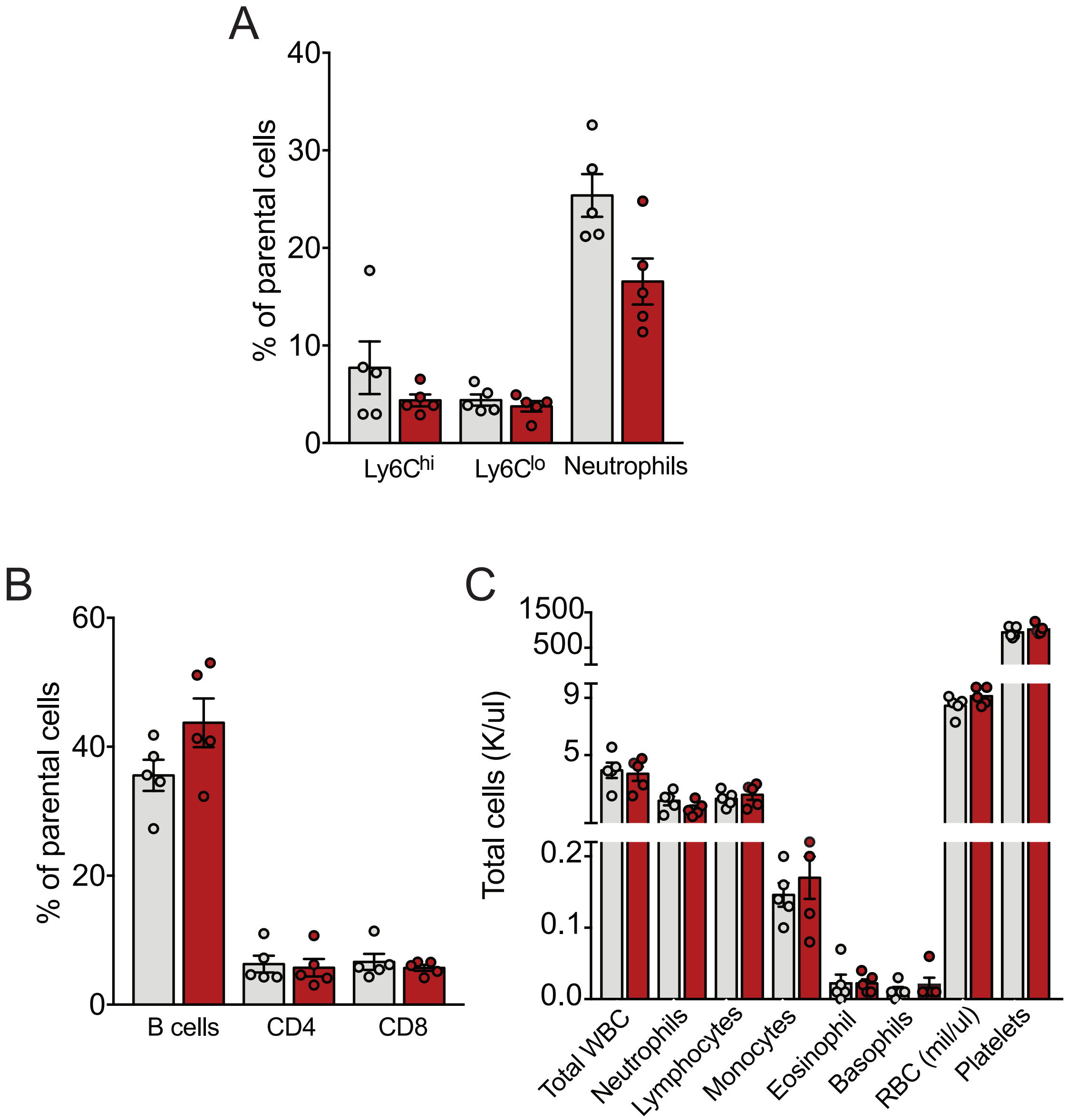
A
% of parental cells
40
30
20
10
0
Ly6Chi
Ly6Clo
Neutrophils
B
% of parental cells
60
40
20
0
B cells
CD4
CD8
C
Total cells (K/ul)
1500
500
9
5
0.2
0.1
0.0
Total WBC
Neutrophils
Lymphocytes
Monocytes
Eosinophil
Basophils
RBC (mil/ul)
Platelets
